# Supplementary material for: A general strategy for recycling polyester wastes into carboxylic acids and hydrocarbons
Source: Nat Commun. 2024 Jan 2;15:160. doi: 10.1038/s41467-023-44604-1 (PMC10761813; doi:10.1038/s41467-023-44604-1)
Supplement: Supplementary file 1 — Supplementary Information [file 41467_2023_44604_MOESM1_ESM.pdf]

# Supplementary Information

## A general strategy for recycling polyester wastes into carboxylic acids and hydrocarbons

Wei Zeng, Yanfei Zhao, Fengtao Zhang, Rongxiang Li, Minhao Tang, Xiaoqian Chang, Ying Wang, Fengtian Wu, Buxing Han, Zhimin Liu*

*Corresponding author: Zhimin Liu, Email: liuzm@iccas.ac.cn.

## This PDF file includes:

Materials
Supplementary Figure 1 to 56

## Materials

Polyglycolic acid (PGA, granule, Mn ~ 150,000), polylactic acid (PLA, powder, Mw ~ 60,000), poly(β-hydroxybutyrate) (PHB, powder, ≥98%) and poly(butylene adipate-co-terephthalate) (PBAT, granule, 50:50, Mn ~ 120,000) were purchased from Macklin Biochemical Co., Ltd. Poly(1,4-butylene adipate) (PBA, granule, Mw ~ 12,000) and poly(D,L-lactide-co-glycolide) (PLGA, granule, 50:50, Mw ~ 15,000-24,000) was from J&K Scientific Co., Ltd. Poly(ethylene terephthalate) (PET, powder, Mw ~ 100,000), poly(butylene terephthalate) (PBT, powder, Mw ~ 100,000), poly(1,4-butylene succinate) (PBS, powder, Mw ~ 100,000) were provided by Hongyuan Polymer Co., Ltd. PET bottle piece was from the Nongfu Spring water plastic bottle. The melting points of all the polyesters are shown in Supplementary Figure 1.

Bromomethane (99%), 3-bromobutyric acid (95%) and benzyl bromide (98%) were purchased from Innochem Scientific & Technology Co., Ltd. Toluene (99%), $NH_4Br$ (99%), methyl benzoate (99.5%), 1,3,5-trimethoxybenzene (99%), 1,3,5-trioxane (99%), sodium chloride (99.5%), methanol (99.5%), 1,2-dibromoethane (99%), dimethyl sulfoxide-d6 (DMSO-d6, 99.8 atom%D) and deuterium oxide ($D_2O$, 99.8 atom%D) were purchased from J&K Scientific Co., Ltd. Pd/C (5 wt% Pd on activated carbon, unreduced) was supplied by Acros Organics.

Acetic acid (99.5%), propionic acid (99%), butyric acid (99%), crotonic acid (98%), 3-butenoic acid (96%) and adipic acid (99%) were purchased from Innochem Scientific & Technology Co., Ltd. Terephthalic acid (99%) and succinic acid (99%) were from J&K Scientific Co., Ltd.

$H_2$ (99.999%) and $N_2$ (99.999%) were provided by Beijing Huayuan Gas Chemical Industry Co., Ltd.

All the ionic liquids (ILs) used were provided by Lanzhou Institute of Chemical Physics (LICP), Chinese Academy of Sciences (CAS), and their chemical structures are shown in Supplementary Figure 2.

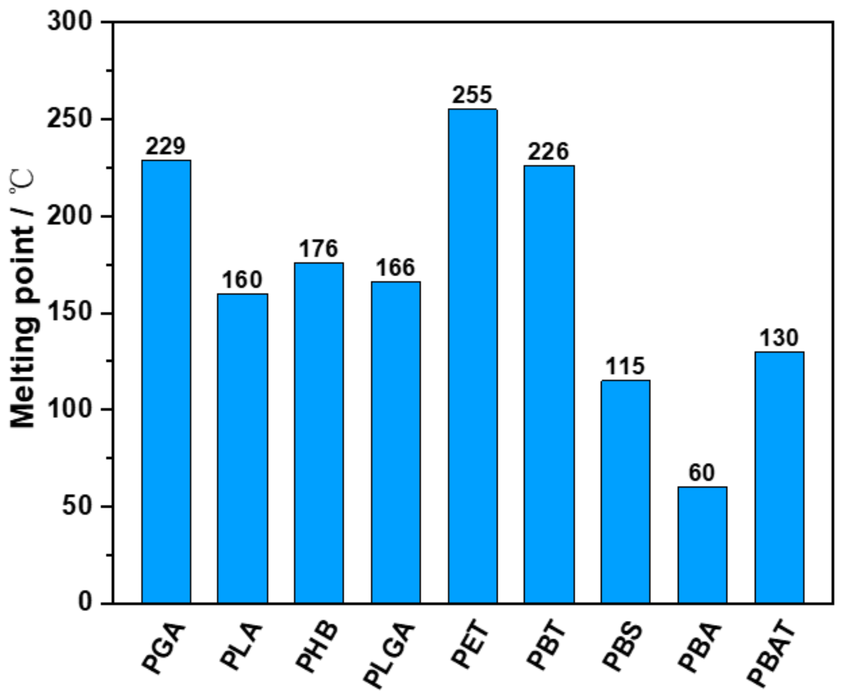


**Supplementary Figure 1. Melting points of the polyesters used in this study.**

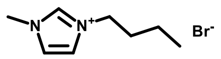

**1-butyl-3-methylimidazolium bromode**
**([BMIm]Br)**

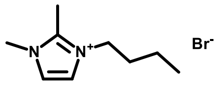

**1-butyl-2,3-dimethylimidazolium bromode**
**([BMMIm]Br)**

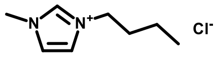

**1-butyl-3-methylimidazolium chloride**
**([BMIm]Cl)**

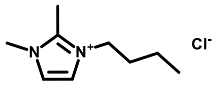

**1-butyl-2,3-dimethylimidazolium chloride**
**([BMMIm]Cl)**

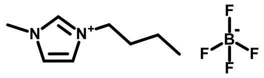

**1-butyl-3-methylimidazolium tetrafluoroborate**
**([BMIm][BF$_4$])**

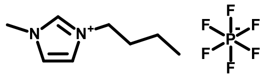

**1-butyl-3-methylimidazolium hexafluorophosphate**
**([BMIm][PF$_6$])**

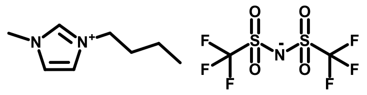

**1-butyl-3-methylimidazolium bis(trifluoromethylsulfonyl)imide**
**([BMIm][NTf$_2$])**

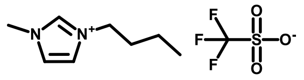

**1-butyl-3-methylimidazolium trifluoromethanesulfonate**
**([BMIm][OTf])**

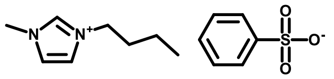

**1-butyl-3-methylimidazolium 4-methylbenzenesulfonate**
**([BMIm][OTs])**

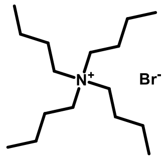

**tetrabutylammonium bromode**
**([N$_{4444}$]Br)**

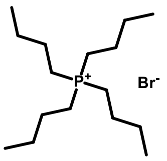

**tetrabutylphosphonium bromode**
**([P$_{4444}$]Br)**

**Supplementary Figure 2. Chemical structures of the ILs used in this study.**

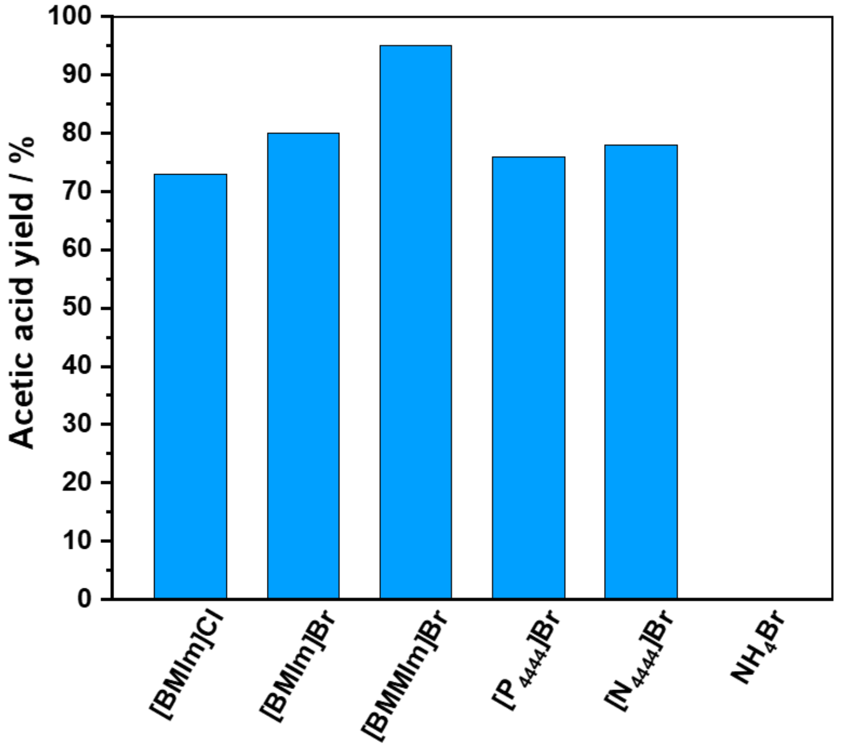


**Supplementary Figure 3. Decomposition of PGA.** Reaction conditions: PGA (1 mmol), IL (2 mmol) or $NH_4Br$ (2 mmol), Pd/C (5 mg, 5 wt% Pd), 5 MPa $H_2$, 180 °C, 24 h. Acetic acid yield was determined by $^1H$ and $^{13}C$ NMR.

**Supplementary Note.** The decomposition of PGA over Pd/C was used to screen the IL catalysts. It was indicated that only the ILs with halide anions such as [BMIm]X, [BMMIm]X, [$N_{4444}$]X and [$P_{4444}$]X (X = Cl, Br) were effective for the degradation of PGA, which afforded acetic acid as the sole product. The reaction did not occur under the solvent-free conditions or in ILs with anions such as $[BF_4]^-$, $[PF_6]^-$, $[NTf_2]^-$, $[OTf]^-$ and $[OTs]^-$. These results suggest that the degradation activity of PGA depends on the nucleophilicity of the ILs. Notably, [BMMIm]Br showed the best performance. Compared to [BMMIm]Br, the C2-H of the [BMIm]Br cation can form hydrogen bond with the $Br^-$ anion, thus reducing the nucleophilicity of the $Br^-$ anion, which may explain why [BMIm]Br showed the lower activity than [BMMIm]Br for the PGA decomposition over Pd/C. Similarly, [BMIm]Cl showed a slightly lower activity than [BMIm]Br, probably due to the hydrogen bond between the C2-H of the IL cation and the IL anion. For comparison, $NH_4Br$ was also examined in the deconstruction of PGA, and it was found to be ineffective. This may be ascribed to the strong electrostatic interaction between its cation and anion, which greatly suppresses the nucleophilicity of the $Br^-$ anion. The above findings demonstrate that the synergistic effect between the IL cation and anion originating from the electrostatic interaction and hydrogen bonding interaction could considerably affect the nucleophilicity of the IL anion. Therefore, it can be concluded that the nucleophilicity of the IL anion itself and the synergistic effect between the IL cation and anion finally determines the catalytic performance of the ILs for the degradation of polyesters.

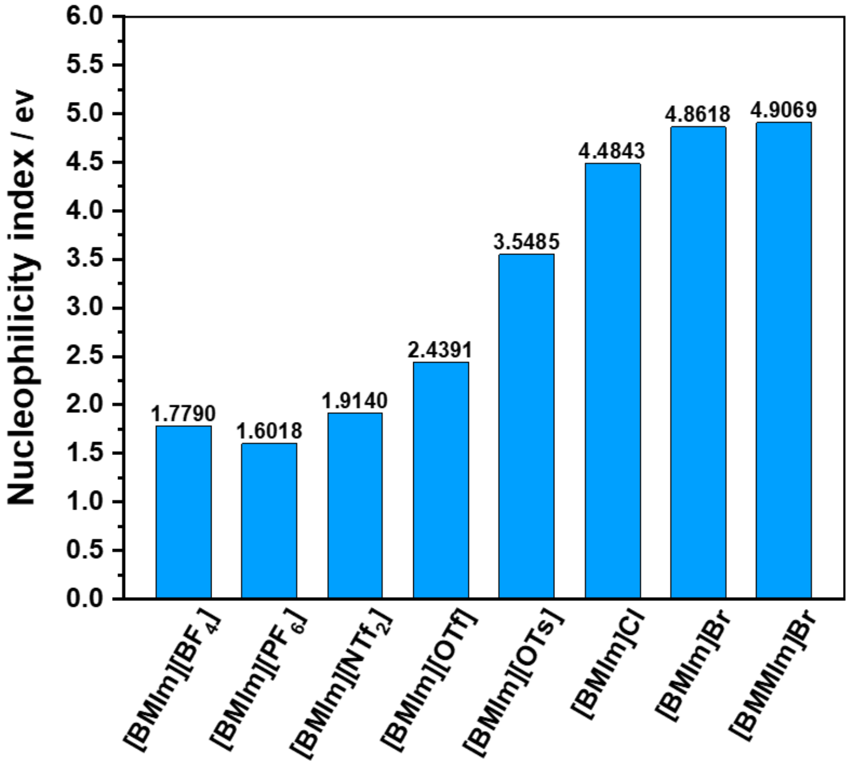


**Supplementary Figure 4. Nucleophilicity index of various ILs.**

**Supplementary Note.** Nucleophilicity index was estimated according to the following equation:

$$\mathbf{N_{(Nu)} = E_{HOMO(Nu)}\ (eV) - E_{HOMO(TCE)}\ (eV)}$$

Tetracyanoethylene (TCE) was taken as a reference due to the lowest HOMO energy in a large series of molecules already considered. For ILs and TCE, geometry optimizations and HOMO calculations were carried out at the M06-2X/def2-TZVP level at 298.15 K. The estimated nucleophilicity index showed that [BMMIm]Br has a higher value, which is quite consistent with its higher catalytic performance from Supplementary Figure 3.

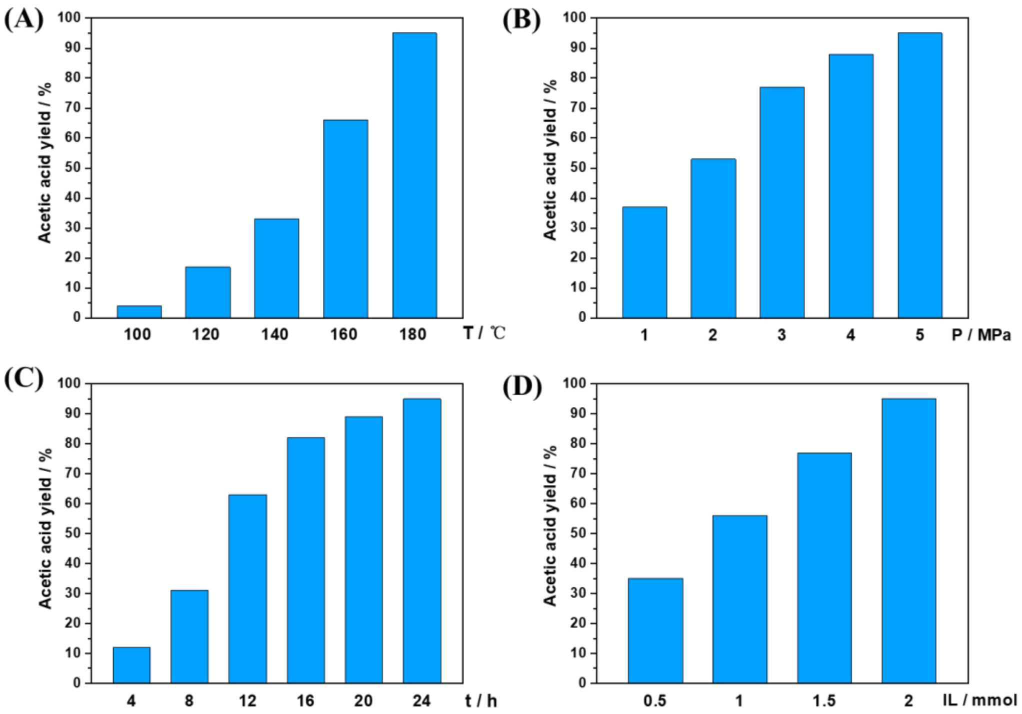


**Supplementary Figure 5. Effects of experimental parameters on the decomposition of PGA. (A)** reaction temperature. **(B)** pressure of $H_2$. **(C)** reaction time. **(D)** amounts of [BMMIm]Br. Reaction conditions: PGA (1 mmol), [BMMIm]Br (2 mmol), Pd/C (5 mg, 5 wt% Pd), 5 MPa $H_2$, 180 °C, 24 h.

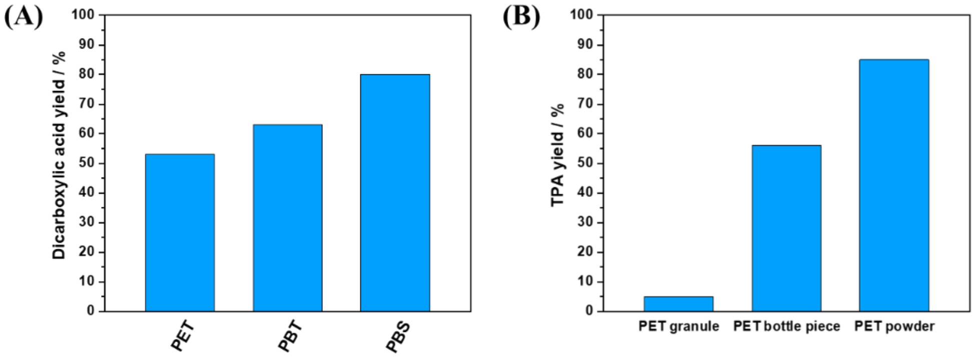


**Supplementary Figure 6. Decomposition of various polyesters.** Reaction conditions: [BMMIm]Br (2 mmol), Pd/C (5 mg, 5 wt% Pd), 5 MPa $H_2$, 180 °C. (**A**) polyester (0.5 mmol, powder, Mw ~ 100,000), 36 h. (**B**) PET (0.5 mmol) with different appearances, 48 h.

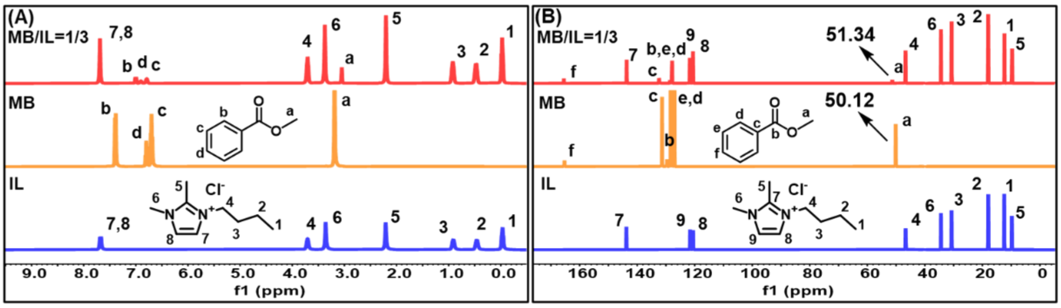


**Supplementary Figure 7. High-temperature NMR spectra of pure [BMMIm]Cl, MB and the MB-[BMMIm]Cl mixture collected at 90 °C.** (**A**) $^{1}$H NMR spectra. (**B**) $^{13}$C NMR spectra.

**Supplementary Note.** The MB-IL mixture with a MB:IL molar ratio of 1:3 was used to perform the $^{1}$H, $^{13}$C and $^{35}$Cl NMR analyses, while the mixture with a molar ratio of 1:1 was used to carry out the $^{17}$O NMR analysis because the $^{17}$O NMR resonance signal was weak. From the $^{13}$C NMR spectra, it is obvious that the resonance signal assigned to the alkoxy C atom in MB shifted from 50.12 to 51.34 ppm as MB mixed with the IL (Supplementary Figure 7B), which indicates that the electron cloud density of this alkoxy C atom decreased due to the hydrogen bonding interaction between MB and IL. This means that this alkoxy C atom becomes more active and easier to be nucleophilically attacked by the halide anion.

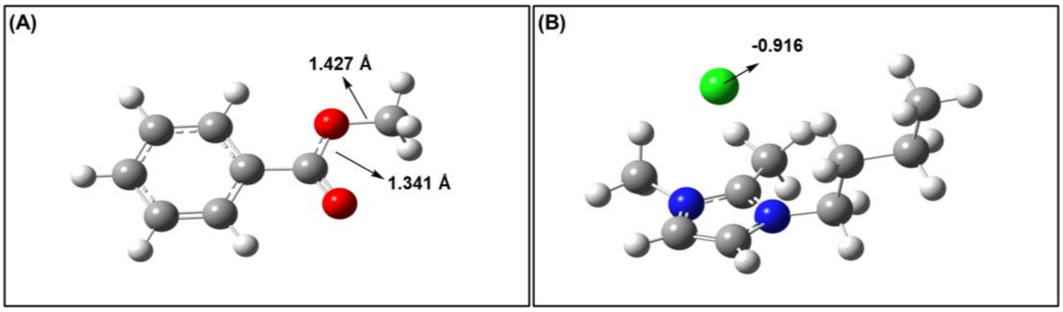


**Supplementary Figure 8. DFT calculations.** (**A**) Optimized geometry of MB. (**B**) Optimized geometry of [BMMIm]Cl. (white ball: H; black ball: C; blue ball: N; green ball: Cl)

**Supplementary Note.** In addition, the estimated $C_{alkoxy}$-O bond length (1.444 Å) of MB in the MB-IL complex becomes longer than that (1.427 Å) in pure MB, while the $C_{acyl}$-O bond length (1.321 Å) becomes shorter than that (1.341 Å) (Fig. 3C and Supplementary Figure 8A). These calculation results suggest that the ester $C_{alkoxy}$-O bond becomes much easier to break due to the hydrogen-bonding interaction between ionic liquid and ester group in polyester.

(a)

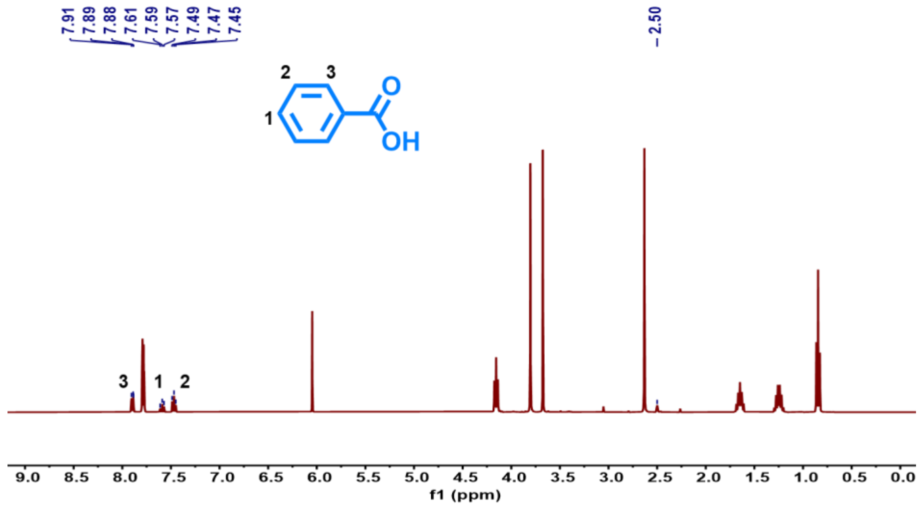


(b)

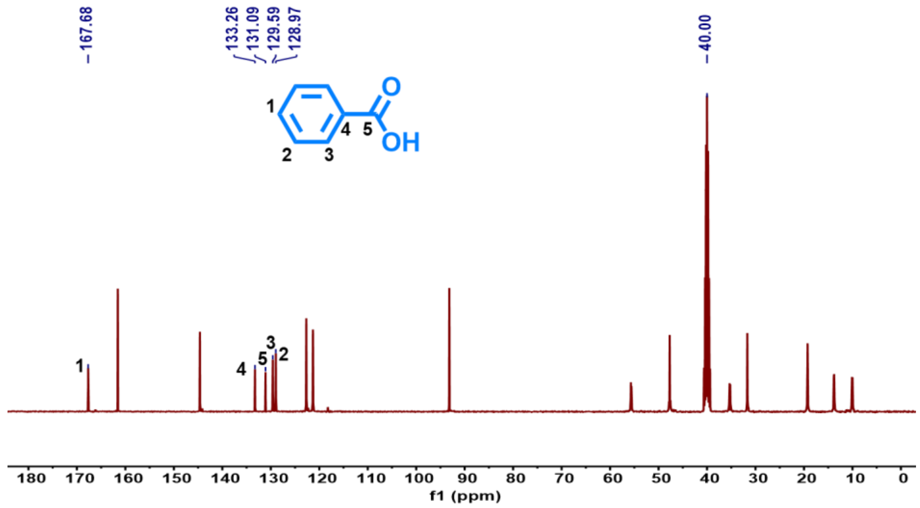


(c)

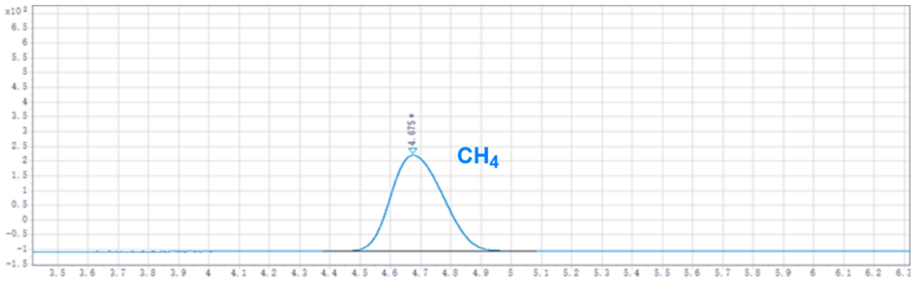


**Supplementary Figure 9. Analysis on control experiments, the reaction of MB over [BMMIm]Br-Pd/C under the $H_2$ atmosphere.** (**a**) $^1$H NMR spectrum (1,3,5-trimethoxybenzene as the internal standard, DMSO-d6, 2.50 ppm) for the liquid reaction solution; (**b**) $^{13}$C NMR spectrum (1,3,5-trimethoxybenzene as the internal standard, DMSO-d6, 40.00 ppm) for the liquid reaction solution; (**c**) GC spectrum of gaseous products.

**Supplementary Note.** These findings confirm the formation of benzoic acid and methane from the reaction of MB over [BMMIm]Br-Pd/C under the $H_2$ atmosphere.

**(a)**

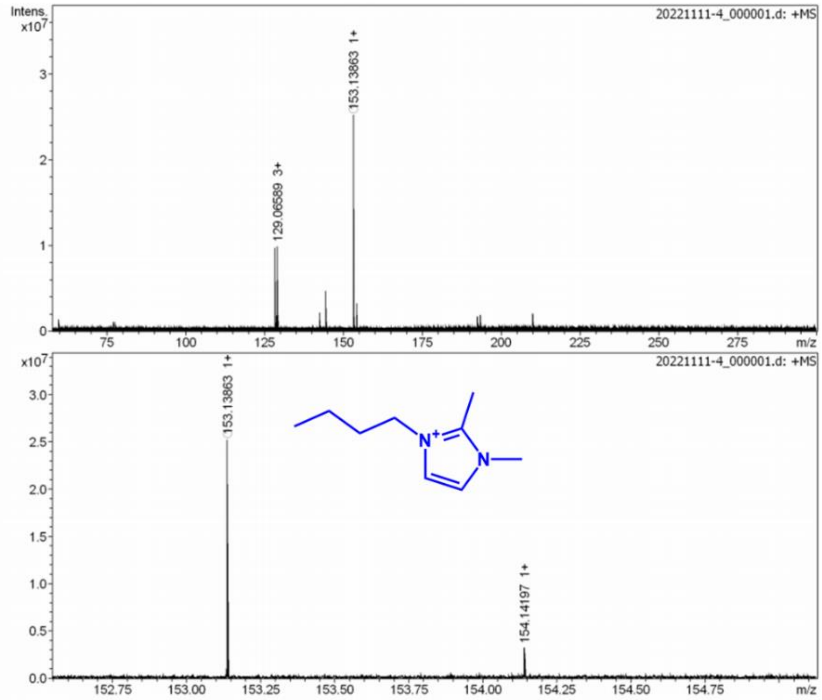


| Meas. m/z | # | Ion Formula | Score | m/z | err [ppm] | Mean err [ppm] | mSigma | rdb | e⁻ Conf | N-Rule |
|---|---|---|---|---|---|---|---|---|---|---|
| 153.138633 | 1 | C9H17N2 | 100.00 | 153.138625 | -0.1 | -0.3 | 14.0 | 2.5 | even | ok |

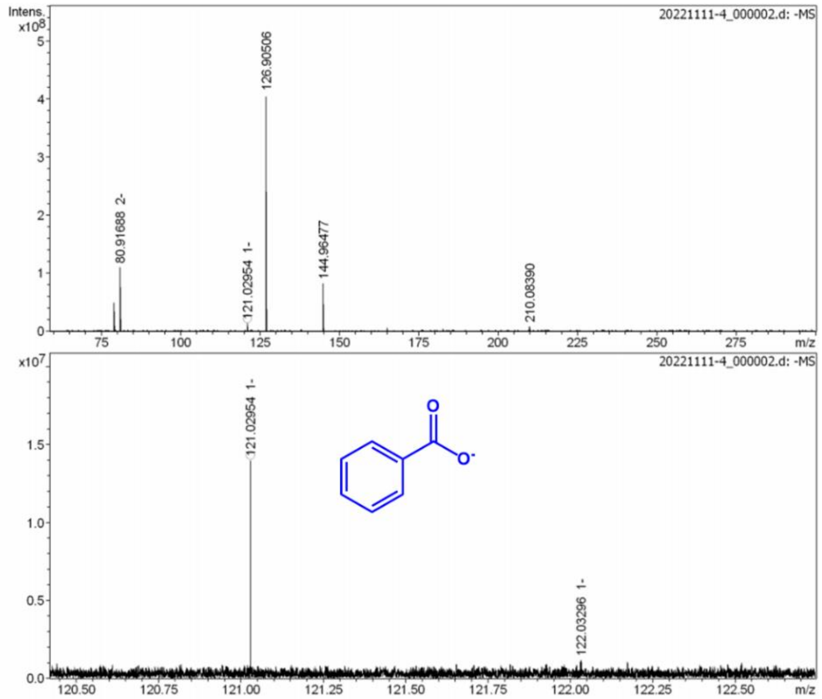


| Meas. m/z | # | Ion Formula | Score | m/z | err [ppm] | Mean err [ppm] | mSigma | rdb | e⁻ Conf | N-Rule |
|---|---|---|---|---|---|---|---|---|---|---|
| 121.029541 | 1 | C7H5O2 | 100.00 | 121.029503 | 0.3 | -0.3 | 5.9 | 5.5 | even | ok |

**(b)**

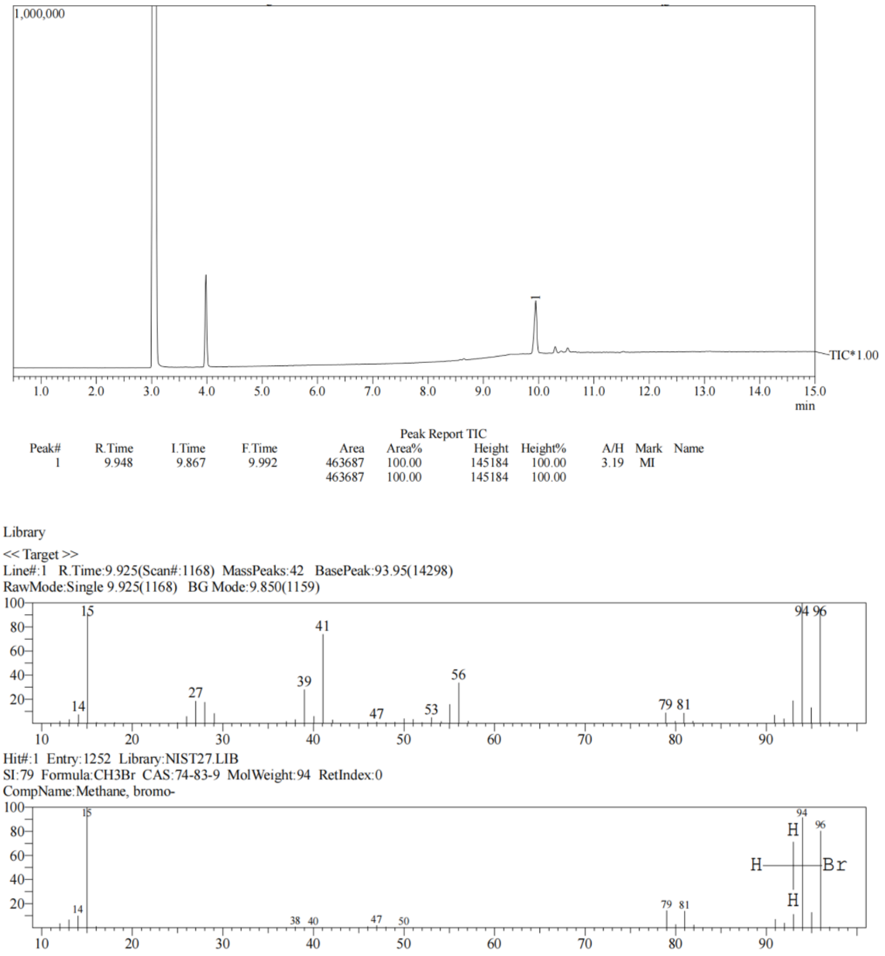


**Supplementary Figure 10. Analysis on control experiments, the reaction of MB over [BMMIm]Br-Pd/C under the $N_2$ atmosphere.** (**a**) HR-ESI-MS spectrum of the liquid reaction solution; (**b**) GC-MS spectrum of the gaseous products.

**Supplementary Note.** These findings confirm the formation of the $[PhCOO]^-$ stabilized by the $[BMMIm]^+$ and methyl bromide from the reaction of MB over [BMMIm]Br-Pd/C under the $N_2$ atmosphere.

**(a)**

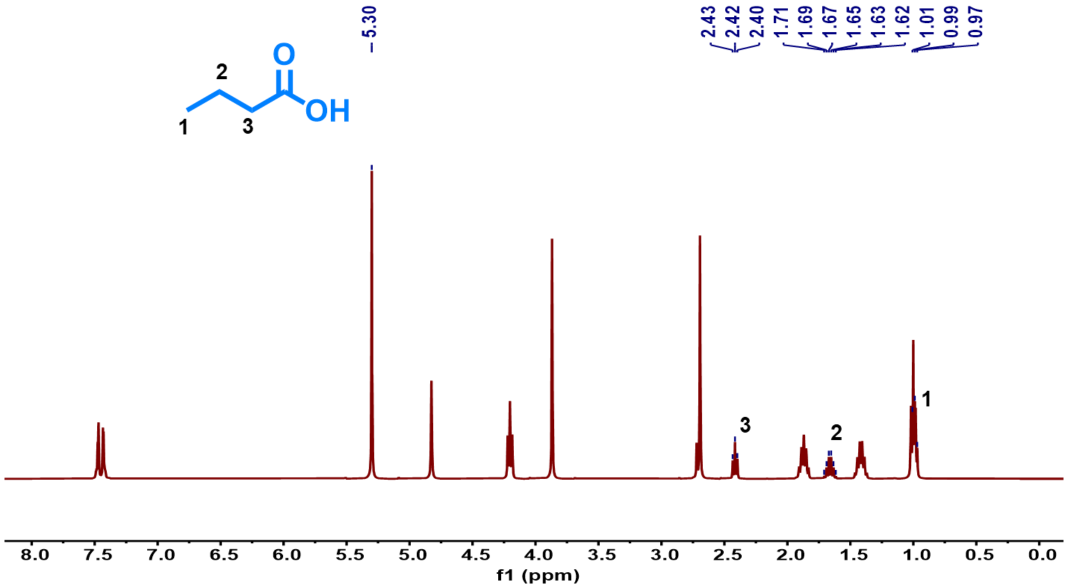


**(b)**

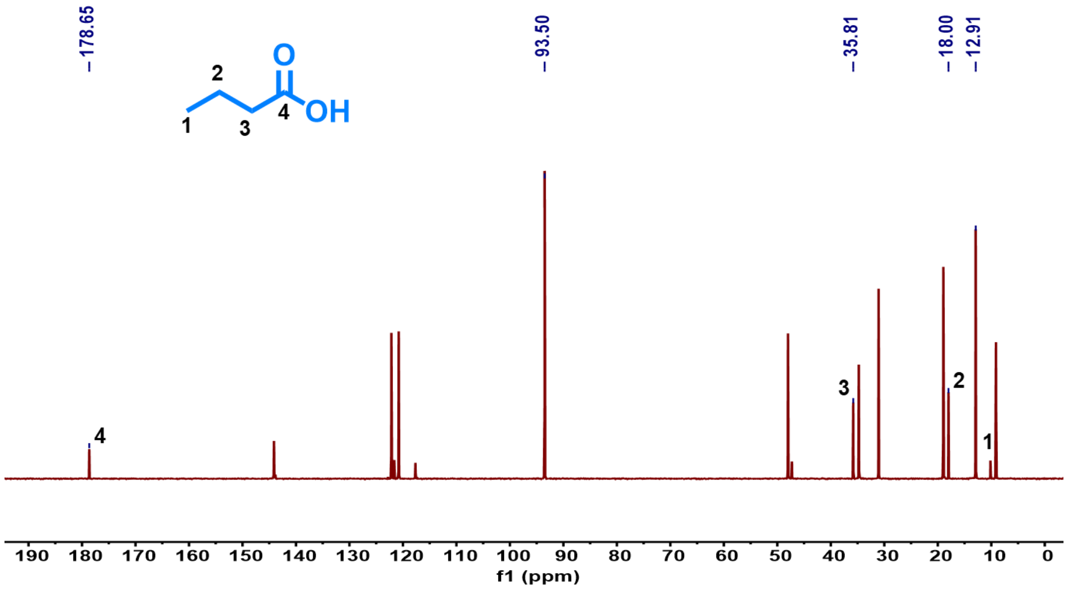


**Supplementary Figure 11. NMR analysis on control experiments, the reaction of 3-bromobutyric acid over [BMMIm]Br-Pd/C under the $H_2$ atmosphere.** (**a**) $^{1}H$ NMR spectrum (1,3,5-trioxane as the internal standard, 5.30 ppm) for the liquid reaction solution; (**b**) $^{13}C$ NMR spectrum (1,3,5- trioxane as the internal standard, 93.50 ppm) for the liquid reaction solution.

**Supplementary Note.** These findings confirm the production of butyric acid from the reaction of 3-bromobutyric acid over [BMMIm]Br-Pd/C under the $H_2$ atmosphere.

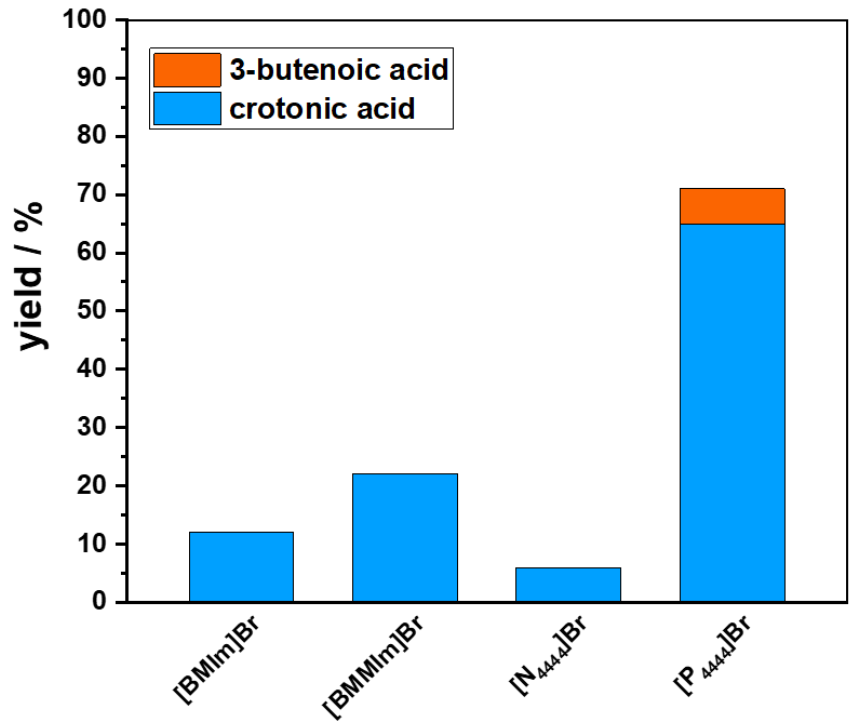


**Supplementary Figure 12. Deconstruction of PHB over various IL catalysts.** Reaction conditions: PHB (1 mmol), IL (2 mmol), 1 MPa $N_2$, 200 °C, 8 h. Crotonic acid and 3-butenoic acid yields were determined by $^1$H and $^{13}$C NMR spectroscopy.

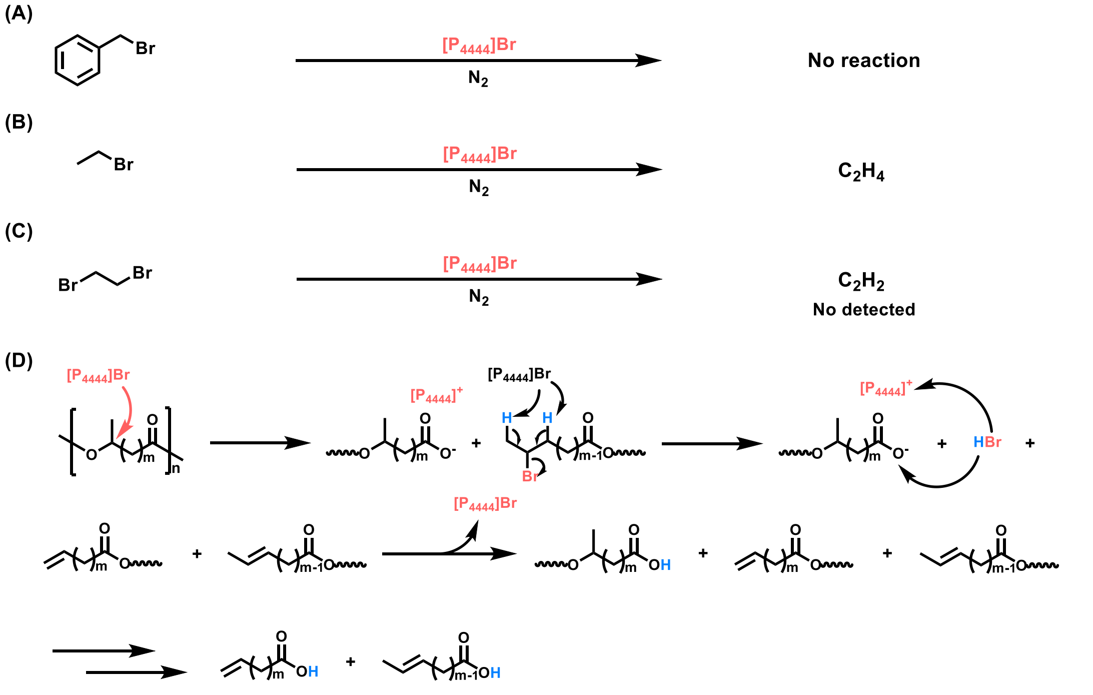


**Supplementary Figure 13. Mechanism study on recycling polyesters with *β*-H over [$P_{4444}$]Br under the $N_2$ atmosphere.** (**A**) The dehalogenation reaction of benzyl bromide over [$P_{4444}$]Br. Reaction conditions: benzyl bromide (1 mmol), [$P_{4444}$]Br (2 mmol), 1 MPa $N_2$, 200 °C, 12 h; (**B**) The dehalogenation reaction of bromoethane over [$P_{4444}$]Br. Reaction conditions: bromoethane (1 mmol), [$P_{4444}$]Br (2 mmol), 1 MPa $N_2$, 200 °C, 12 h; (**C**) The dehalogenation reaction of 1,2-dibromoethane over [$P_{4444}$]Br. Reaction conditions: 1,2-dibromoethane (0.5 mmol), [$P_{4444}$]Br (2 mmol), 1 MPa $N_2$, 200 °C, 12 h; (**D**) Possible reaction pathway for the degradation of polyesters with *β*-H over [$P_{4444}$]Br under the $N_2$ atmosphere. m≥1.

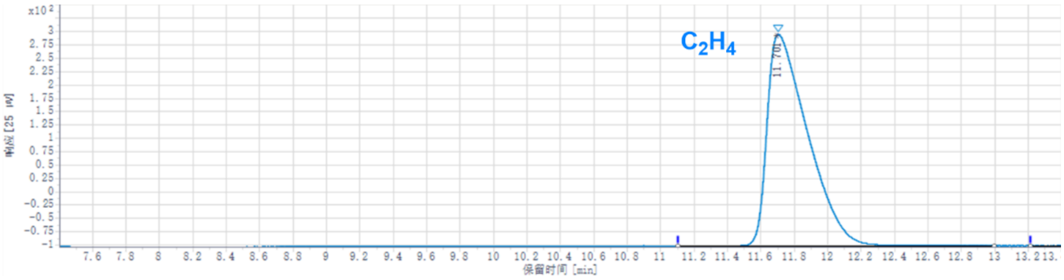


**Supplementary Figure 14. GC spectrum of the gaseous products from the dehalogenation reaction of bromoethane over [$P_{4444}$]Br under the $N_2$ atmosphere.**

(a)

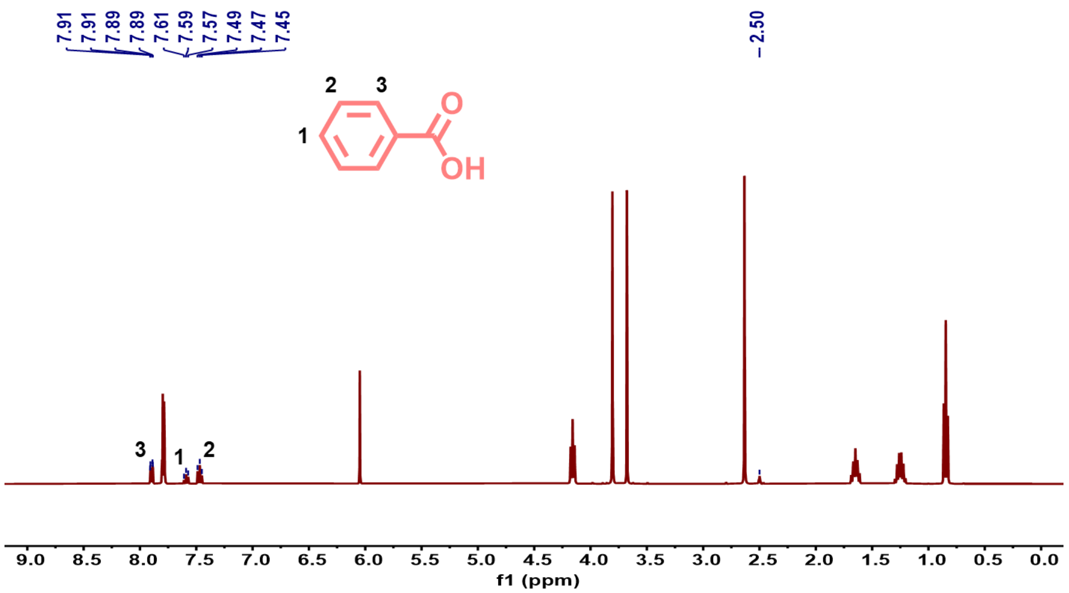


(b)

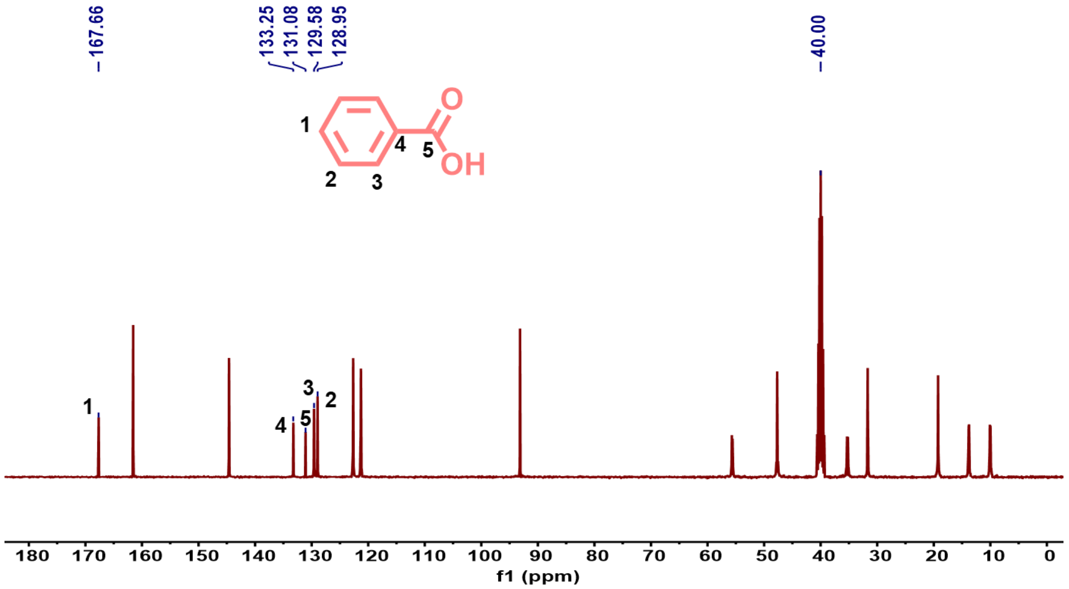


**Supplementary Figure 15. NMR spectra of commercial benzoic acid in [BMMIm]Br.** (**a**) $^{1}H$ NMR spectrum (1,3,5-trimethoxybenzene as the internal standard, DMSO-d6, 2.50 ppm); (**b**) $^{13}C$ NMR spectrum (1,3,5-trimethoxybenzene as the internal standard, DMSO-d6, 40.00 ppm).

(a)

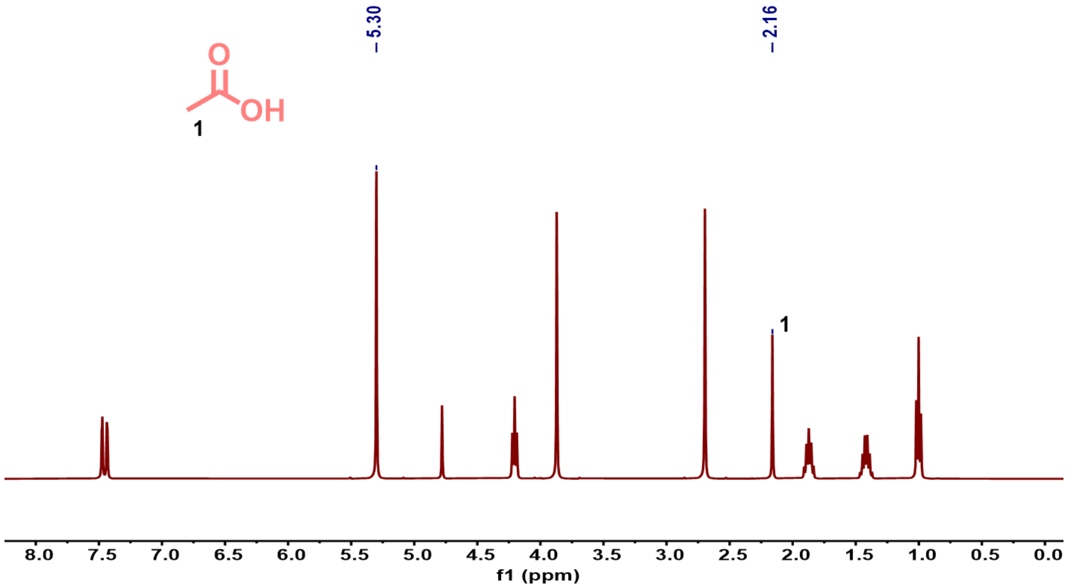


(b)

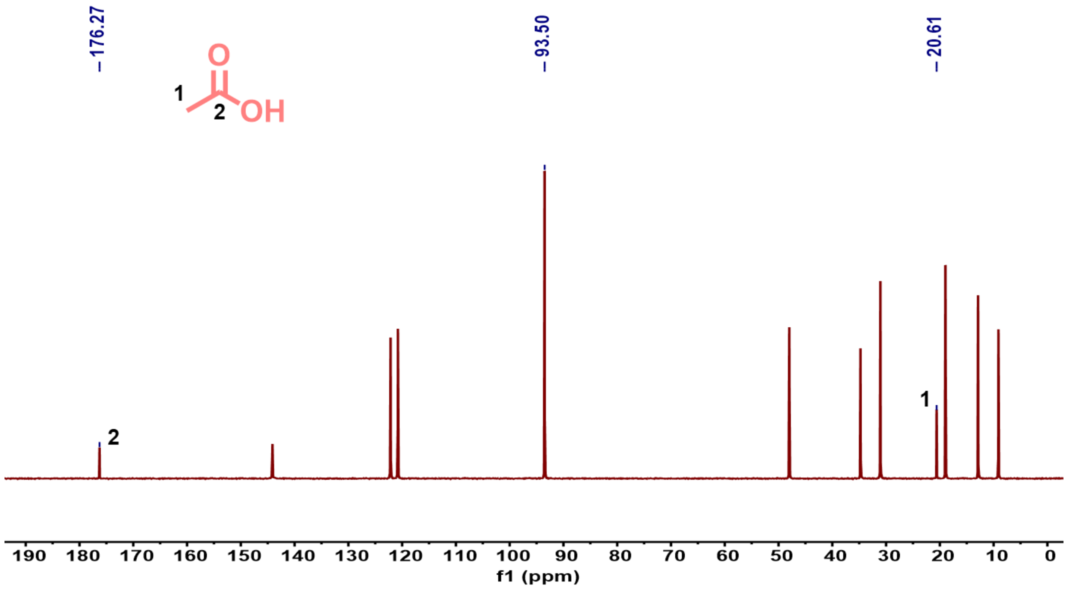


**Supplementary Figure 16. NMR spectra of commercial acetic acid in [BMMIm]Br.** (**a**) $^{1}H$ NMR spectrum (1,3,5-trioxane as the internal standard, 5.30 ppm); (**b**) $^{13}C$ NMR spectrum (1,3,5- trioxane as the internal standard, 93.50 ppm).

(a)

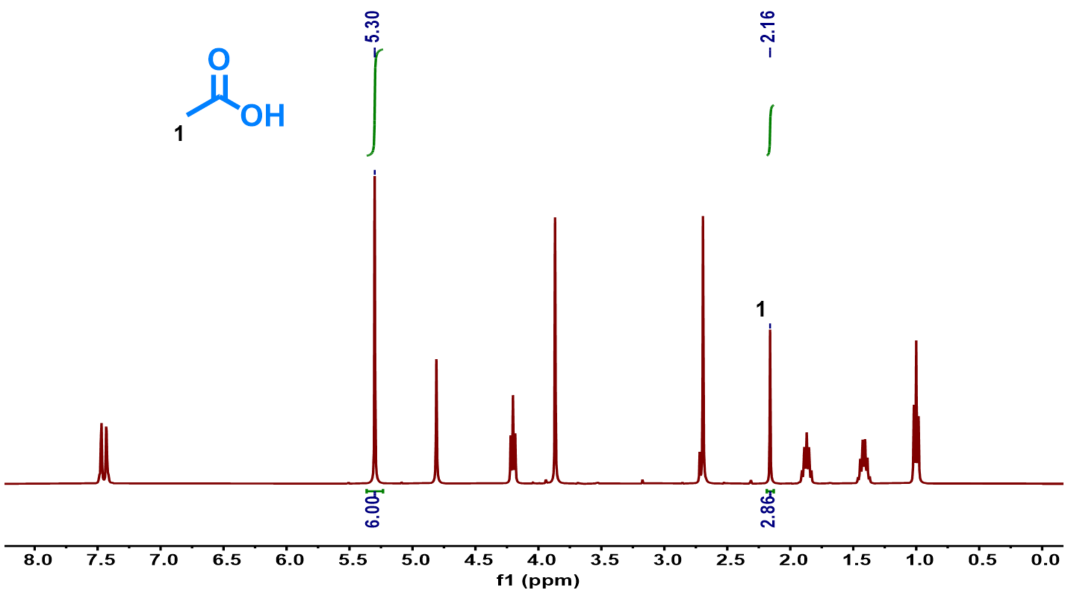


(b)

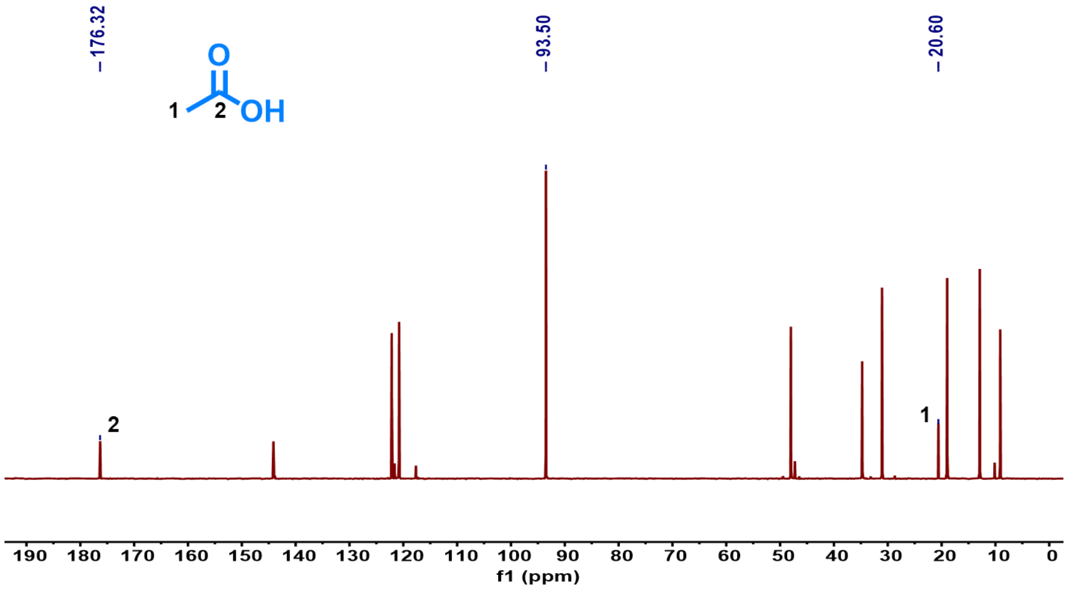


**Supplementary Figure 17. NMR spectra of the liquid reaction solution from PGA granule decomposition over [BMMIm]Br-Pd/C under the $H_2$ atmosphere.** (**a**) $^{1}$H NMR spectrum (1,3,5-trioxane as the internal standard, 5.30 ppm); (**b**) $^{13}$C NMR spectrum (1,3,5- trioxane as the internal standard, 93.50 ppm).

(a)

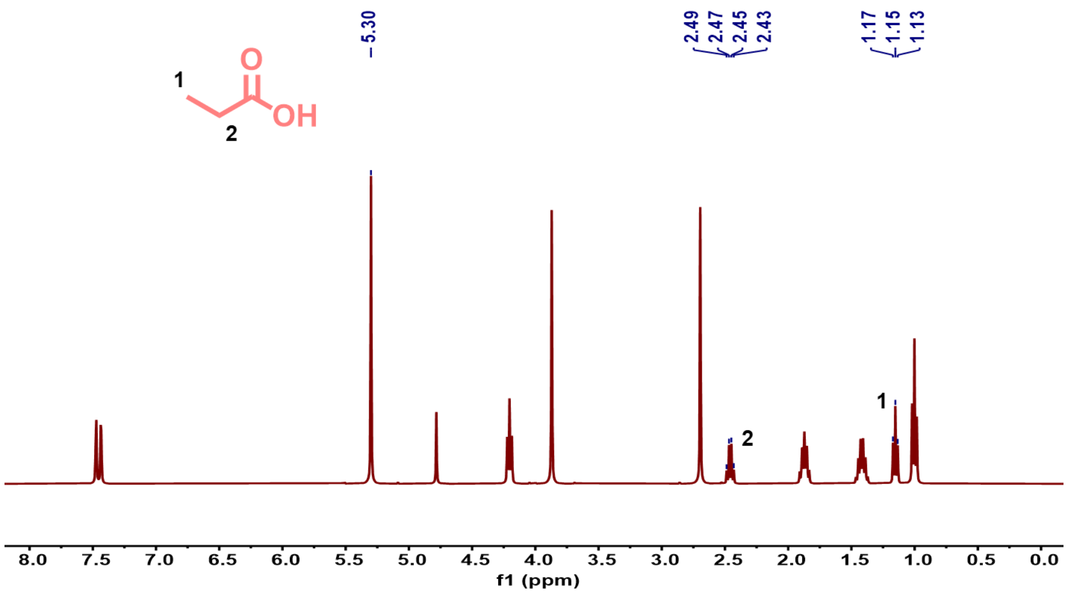


(b)

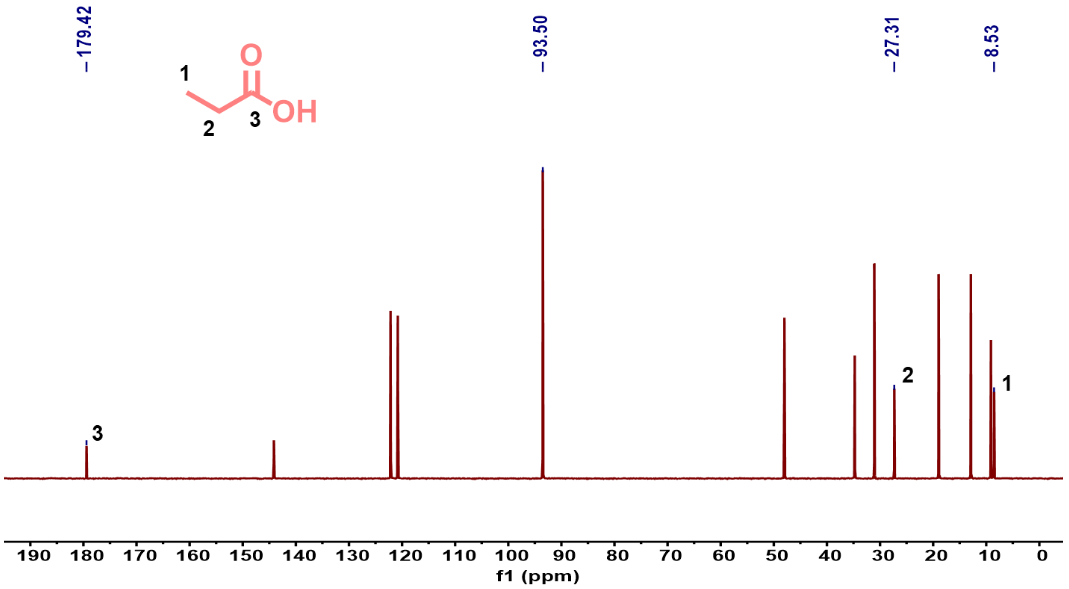


**Supplementary Figure 18. NMR spectra of commercial propionic acid in [BMMIm]Br.** (**a**) $^{1}$H NMR spectrum (1,3,5-trioxane as the internal standard, 5.30 ppm); (**b**) $^{13}$C NMR spectrum (1,3,5- trioxane as the internal standard, 93.50 ppm).

(a)

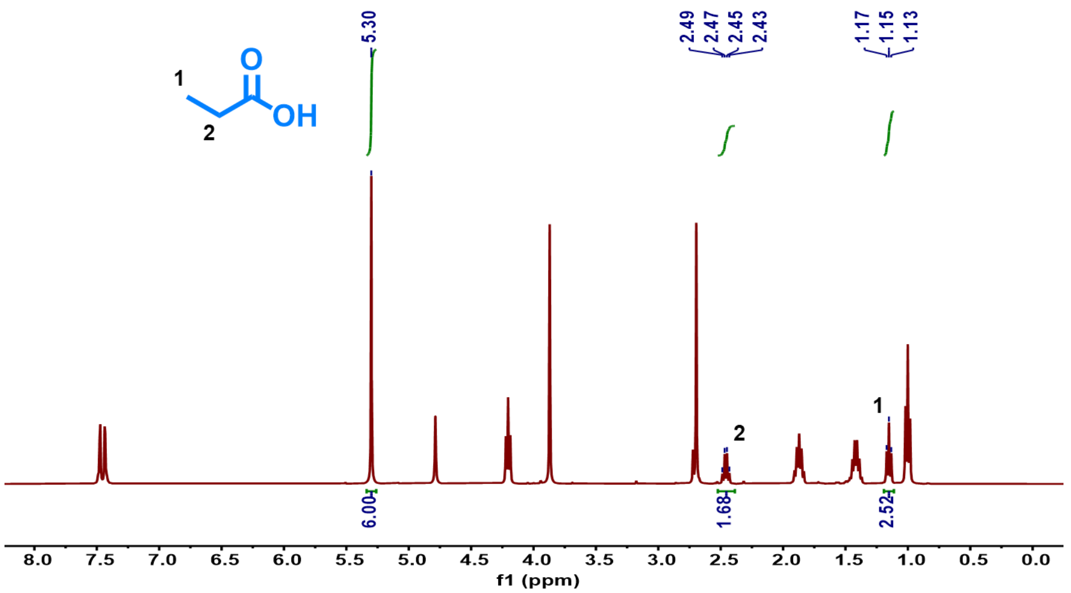


(b)

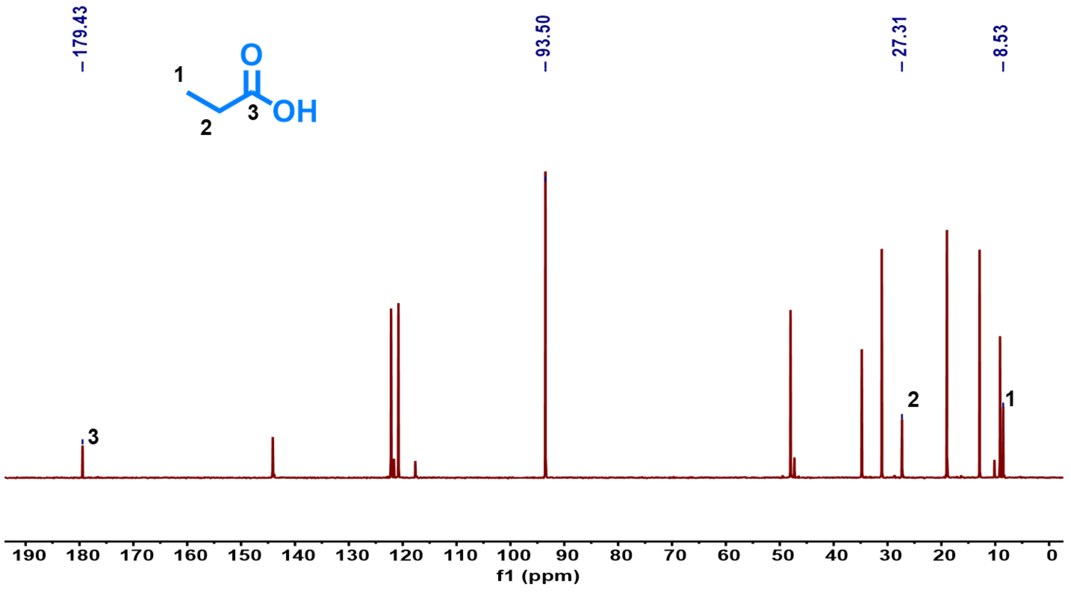


**Supplementary Figure 19. NMR spectra of the liquid reaction solution from PLA powder decomposition over [BMMIm]Br-Pd/C under the $H_2$ atmosphere.** (**a**) $^{1}$H NMR spectrum (1,3,5-trioxane as the internal standard, 5.30 ppm); (**b**) $^{13}$C NMR spectrum (1,3,5- trioxane as the internal standard, 93.50 ppm).

(a)

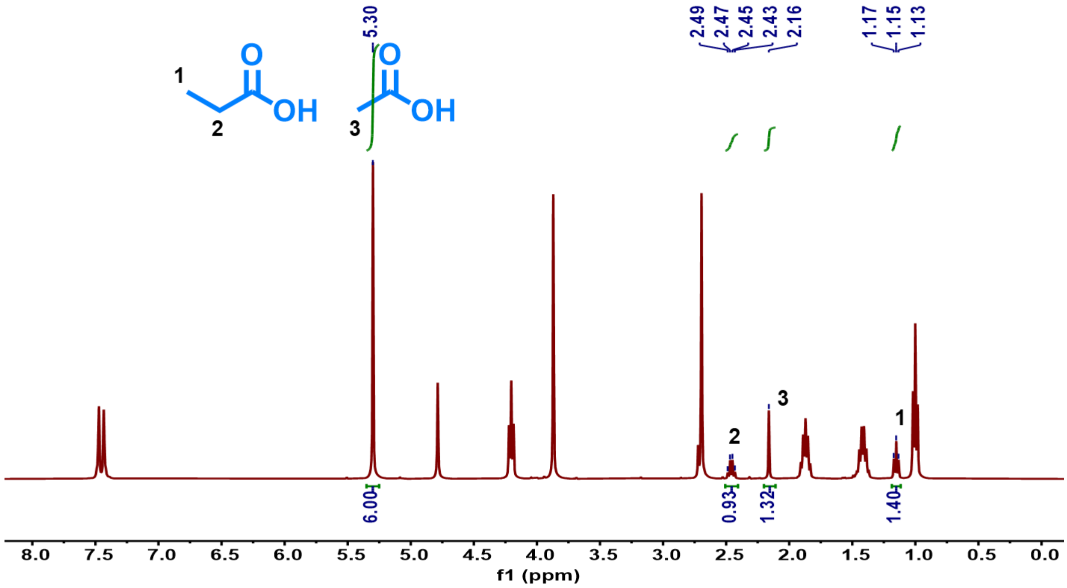


(b)

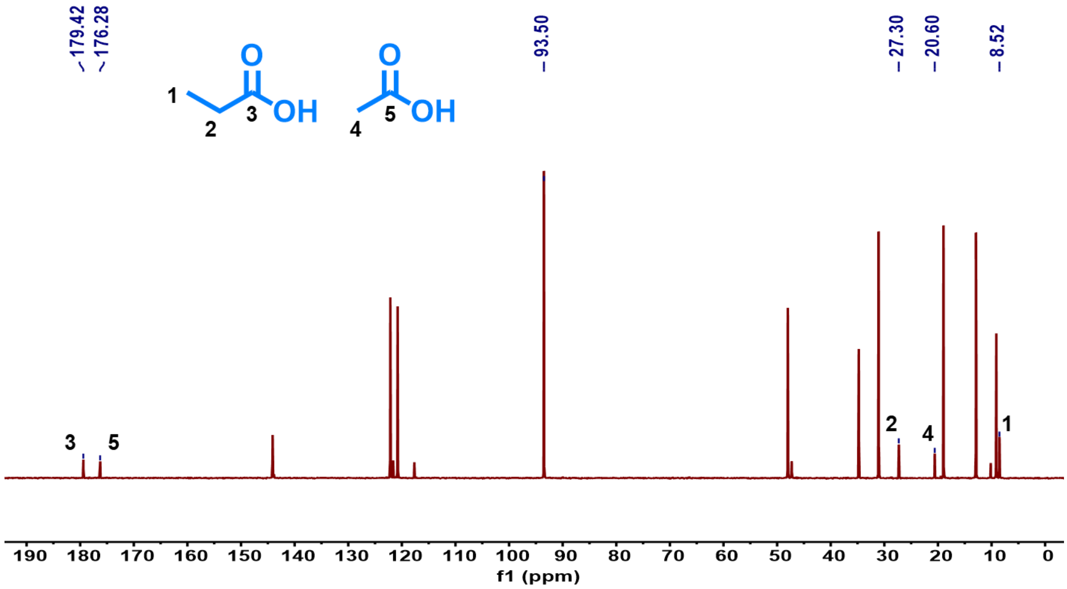


**Supplementary Figure 20. NMR spectra of the liquid reaction solution from PLGA granule decomposition over [BMMIm]Br-Pd/C under the $H_2$ atmosphere.** (**a**) $^{1}H$ NMR spectrum (1,3,5-trioxane as the internal standard, 5.30 ppm); (**b**) $^{13}C$ NMR spectrum (1,3,5- trioxane as the internal standard, 93.50 ppm).

(a)

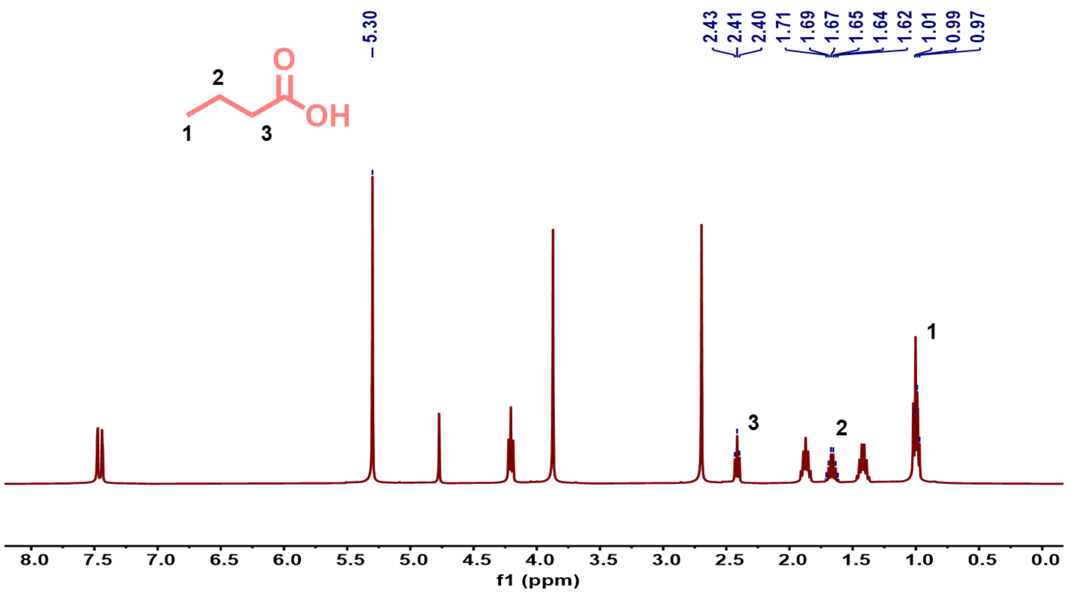


(b)

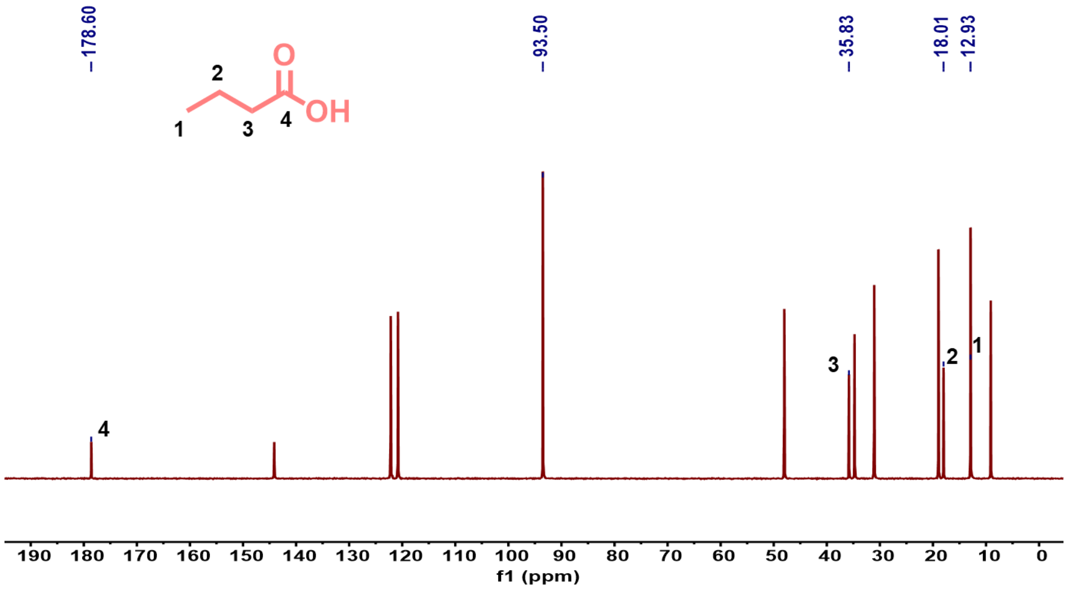


**Supplementary Figure 21. NMR spectra of commercial butyric acid in [BMMIm]Br.** (**a**) $^{1}$H NMR spectrum (1,3,5-trioxane as the internal standard, 5.30 ppm); (**b**) $^{13}$C NMR spectrum (1,3,5- trioxane as the internal standard, 93.50 ppm).

(a)

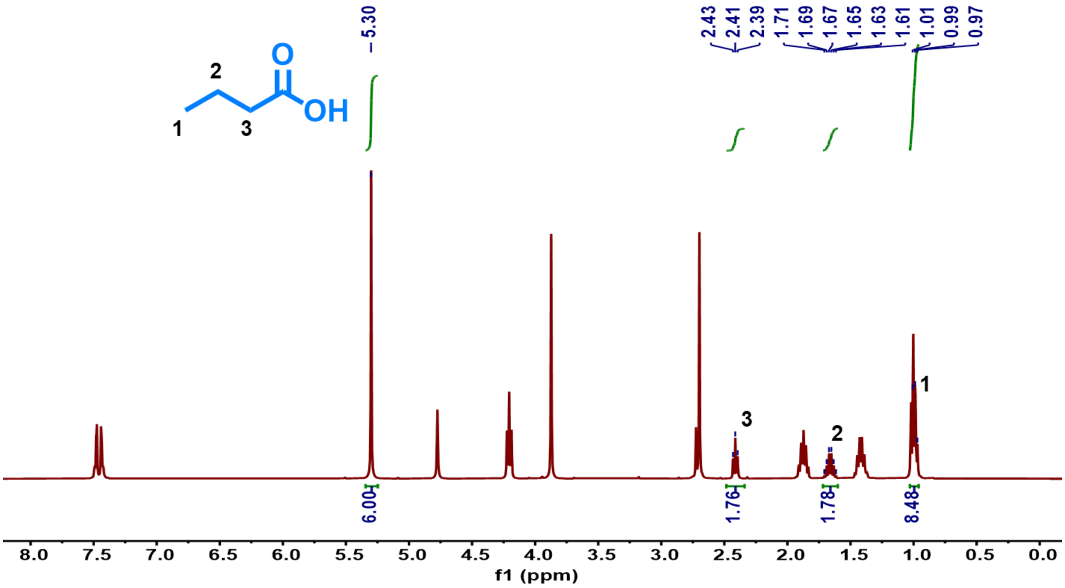


(b)

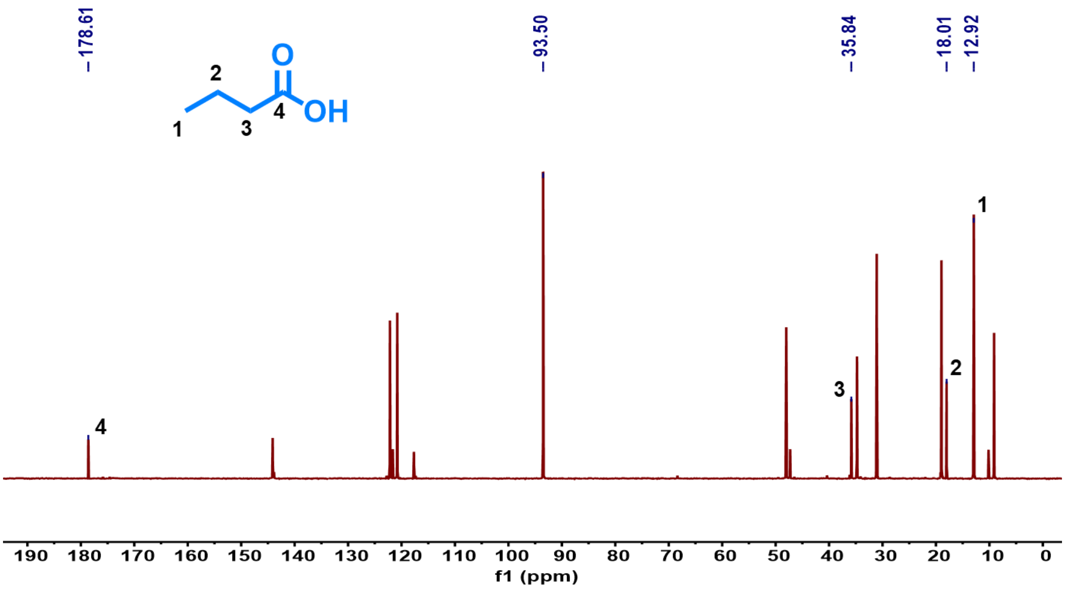


**Supplementary Figure 22. NMR spectra of the liquid reaction solution from PHB powder decomposition over [BMMIm]Br-Pd/C under the $H_2$ atmosphere.** (**a**) $^{1}H$ NMR spectrum (1,3,5-trioxane as the internal standard, 5.30 ppm); (**b**) $^{13}C$ NMR spectrum (1,3,5- trioxane as the internal standard, 93.50 ppm).

**(a)**

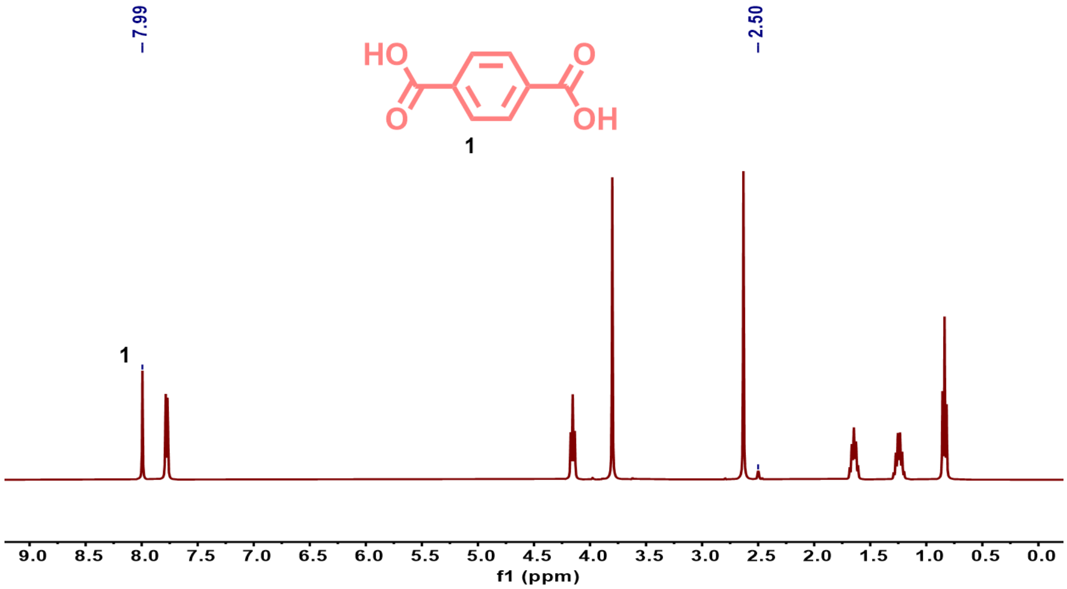


**(b)**

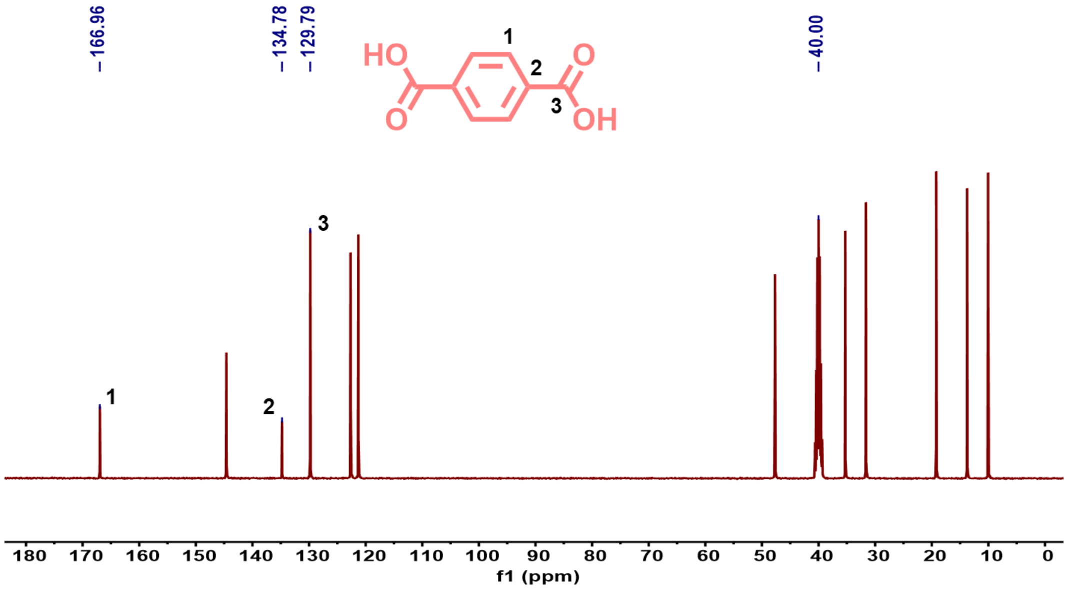


**Supplementary Figure 23. NMR spectra of commercial terephthalic acid in [BMMIm]Br.** (**a**) $^{1}H$ NMR spectrum (DMSO-d6, 2.50 ppm); (**b**) $^{13}C$ NMR spectrum (DMSO-d6, 40.00 ppm).

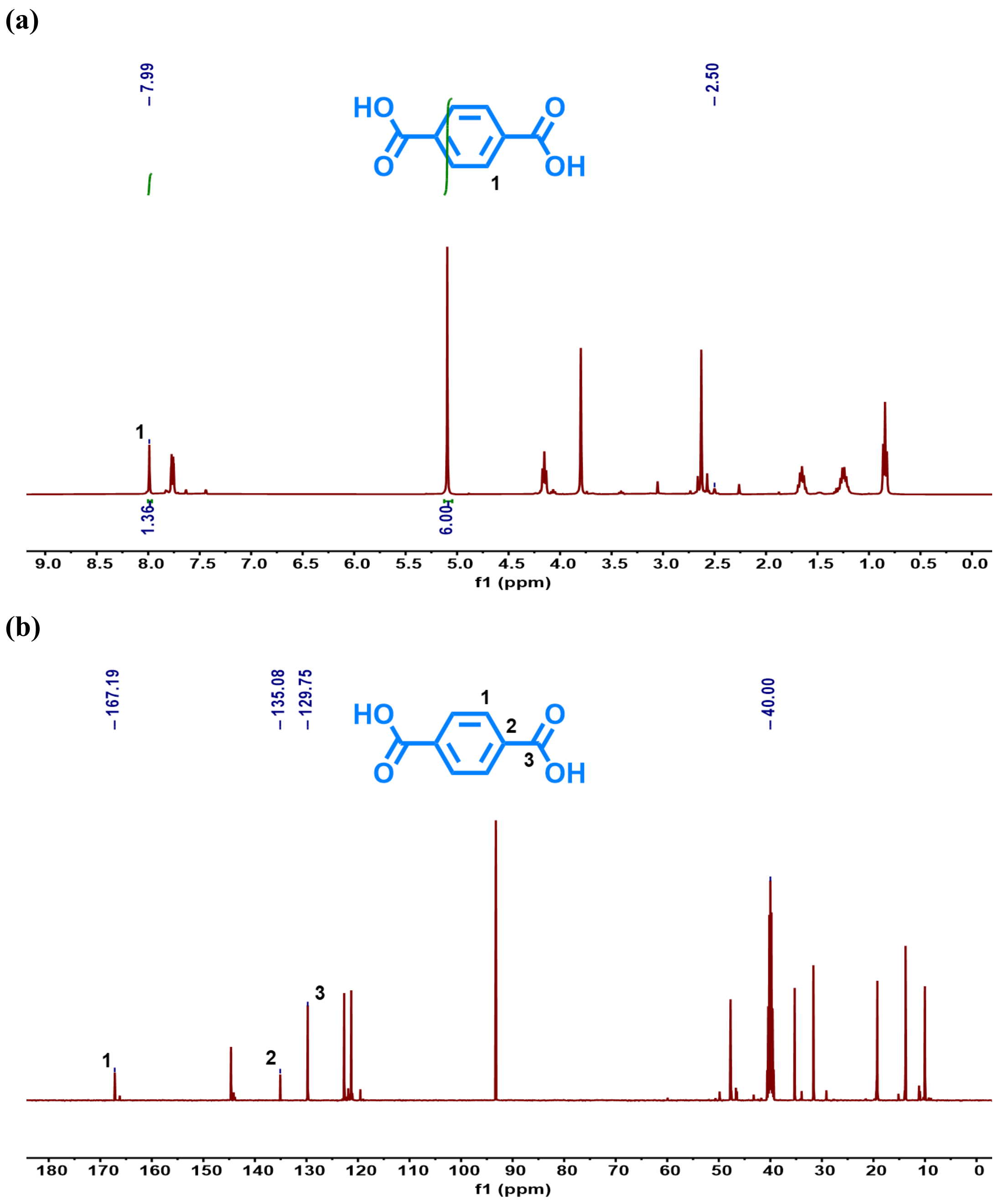


**Supplementary Figure 24. NMR spectra of the liquid reaction solution from PET powder decomposition over [BMMIm]Br-Pd/C under the $H_2$ atmosphere.** (**a**) $^{1}H$ NMR spectrum (1,3,5-trioxane as the internal standard, DMSO-d6, 2.50 ppm); (**b**) $^{13}C$ NMR spectrum (1,3,5-trioxane as the internal standard, DMSO-d6, 40.00 ppm).

**(a)**

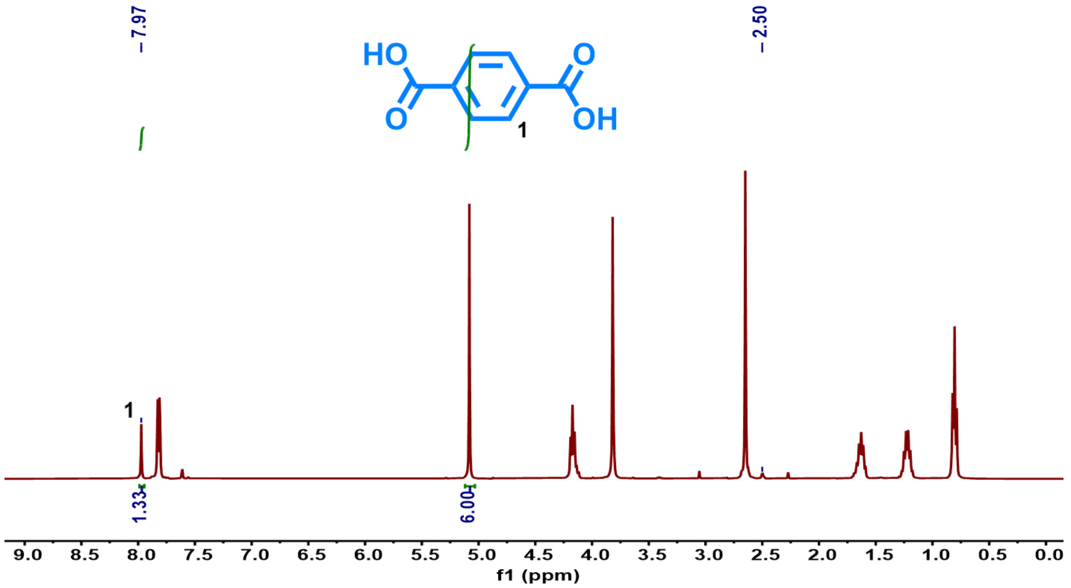


**(b)**

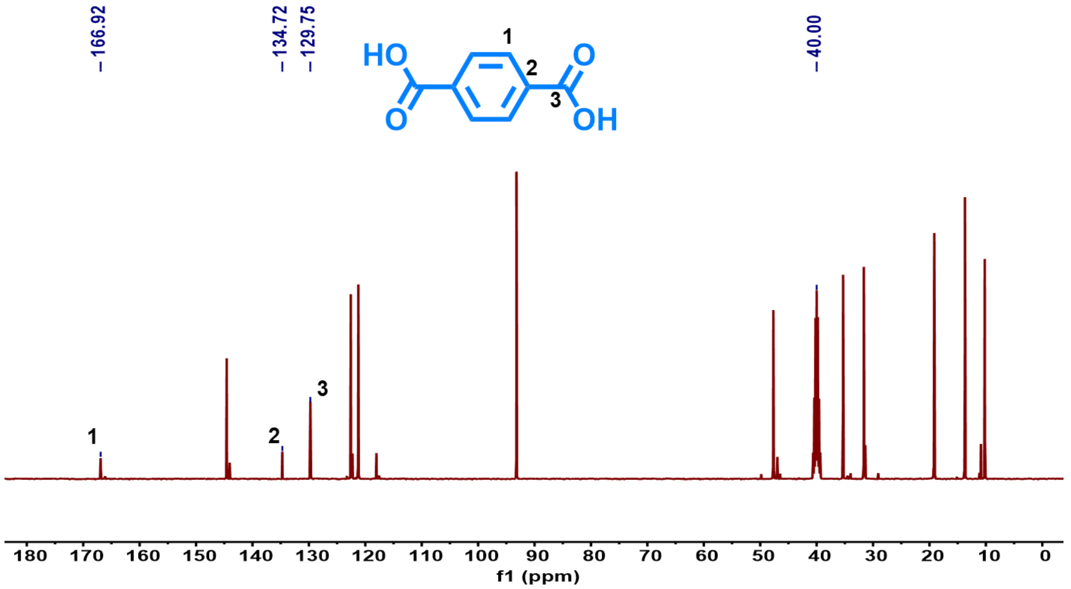


**Supplementary Figure 25. NMR spectra of the liquid reaction solution from decomposition of PET piece from plastic bottle over [BMMIm]Br-Pd/C under the $H_2$ atmosphere.** (**a**) $^{1}H$ NMR spectrum (1,3,5-trioxane as the internal standard, DMSO-d6, 2.50 ppm); (**b**) $^{13}C$ NMR spectrum (1,3,5-trioxane as the internal standard, DMSO-d6, 40.00 ppm).

(a)

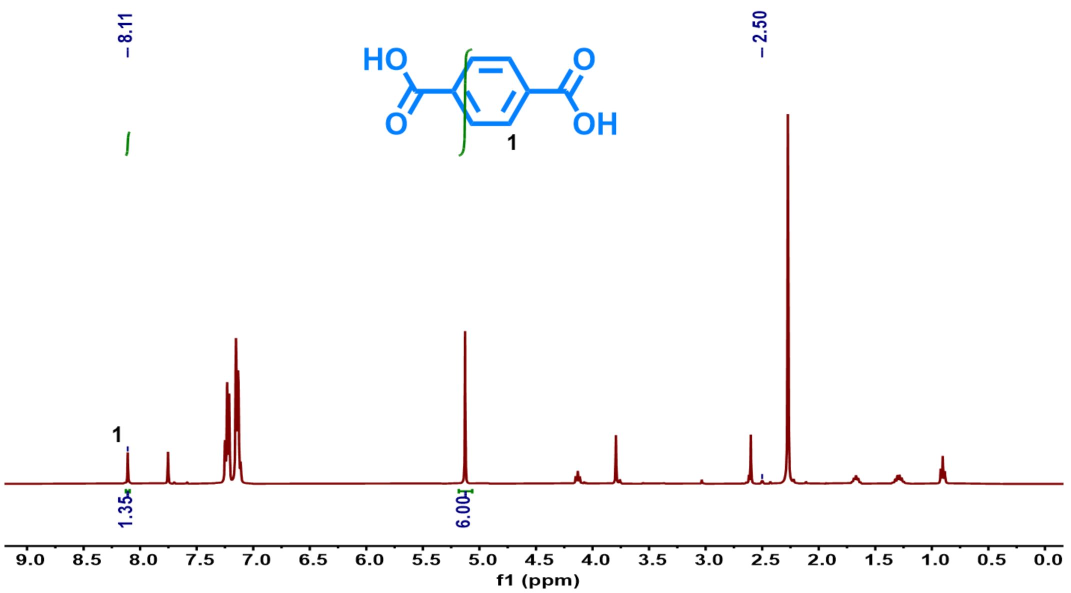


(b)

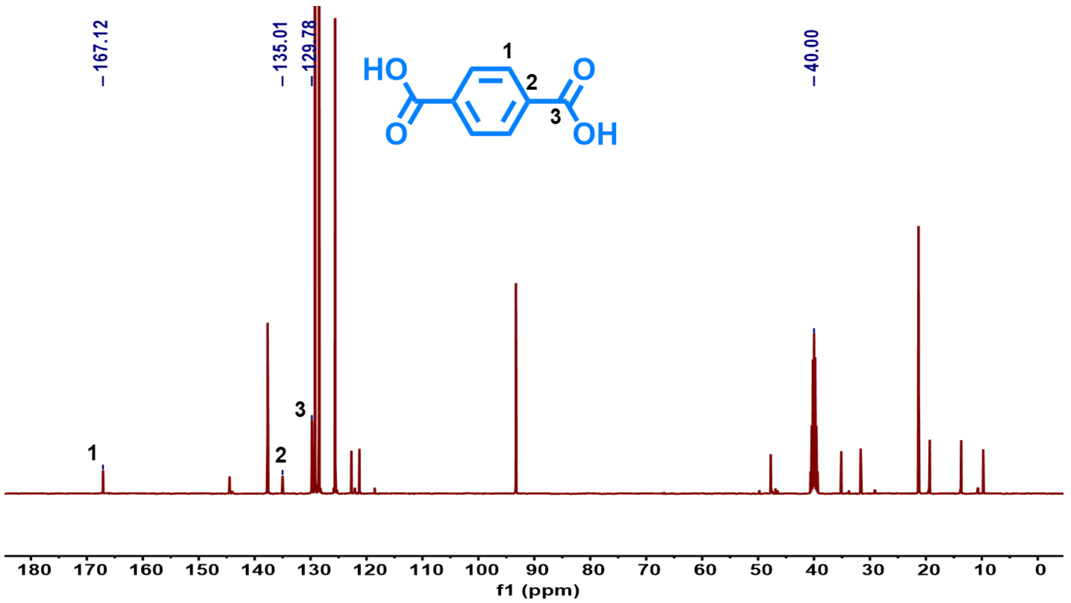


**Supplementary Figure 26. NMR spectra of the liquid reaction solution from decomposition of PET piece from plastic bottle over [BMMIm]Br-Pd/C in toluene under the $H_2$ atmosphere.** (**a**) $^1$H NMR spectrum (1,3,5-trioxane as the internal standard, DMSO-d6, 2.50 ppm); (**b**) $^{13}$C NMR spectrum (1,3,5-trioxane as the internal standard, DMSO-d6, 40.00 ppm).

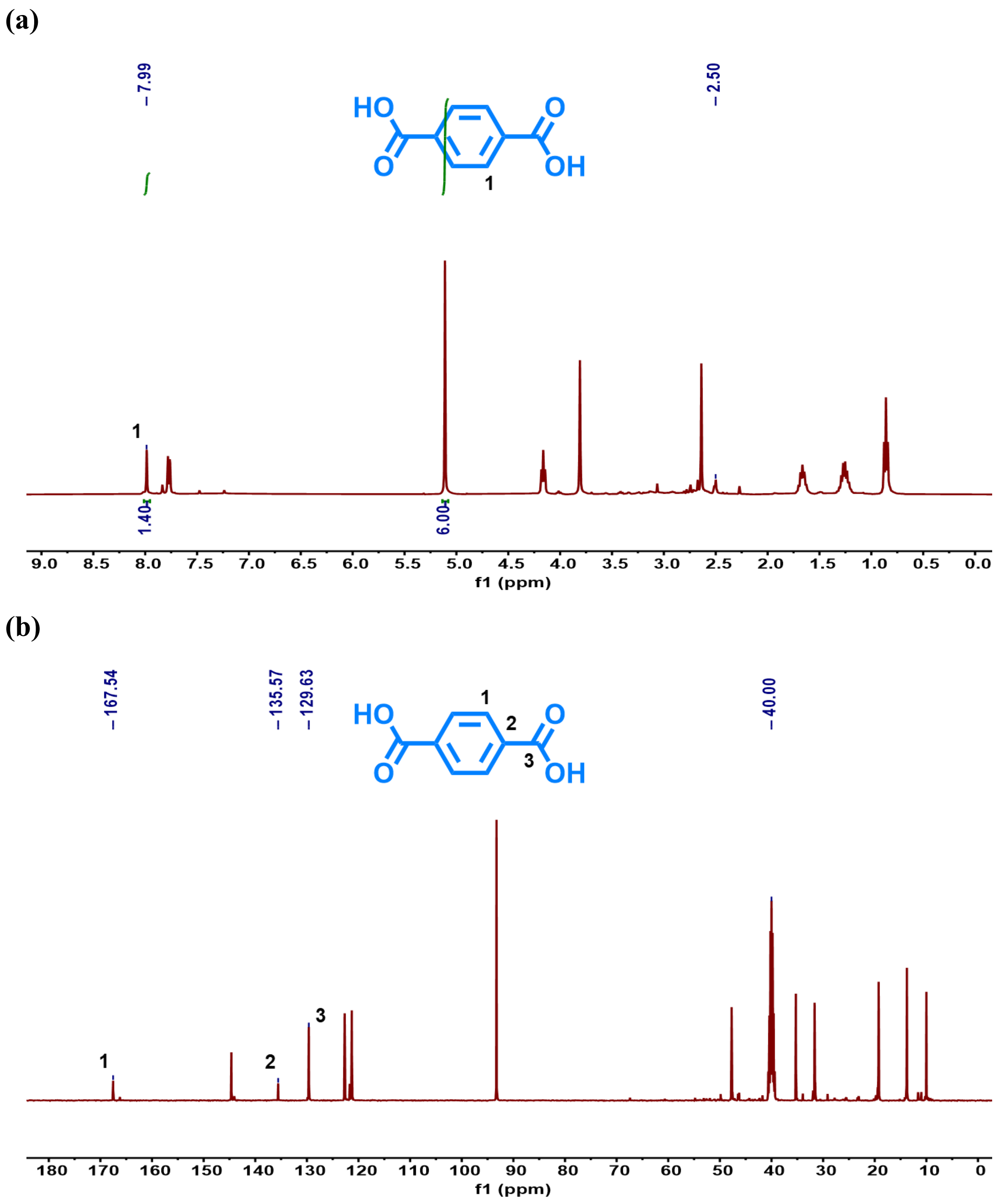


**Supplementary Figure 27. NMR spectra of the liquid reaction solution from PBT powder decomposition over [BMMIm]Br-Pd/C under the $H_2$ atmosphere.** (**a**) $^{1}H$ NMR spectrum (1,3,5-trioxane as the internal standard, DMSO-d6, 2.50 ppm); (**b**) $^{13}C$ NMR spectrum (1,3,5-trioxane as the internal standard, DMSO-d6, 40.00 ppm).

(a)

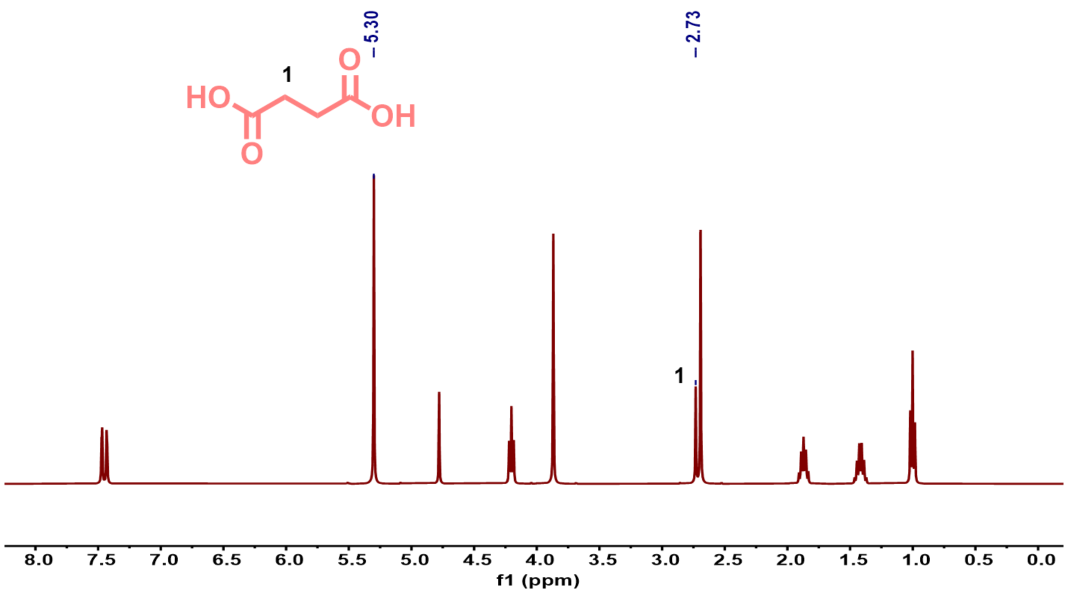


(b)

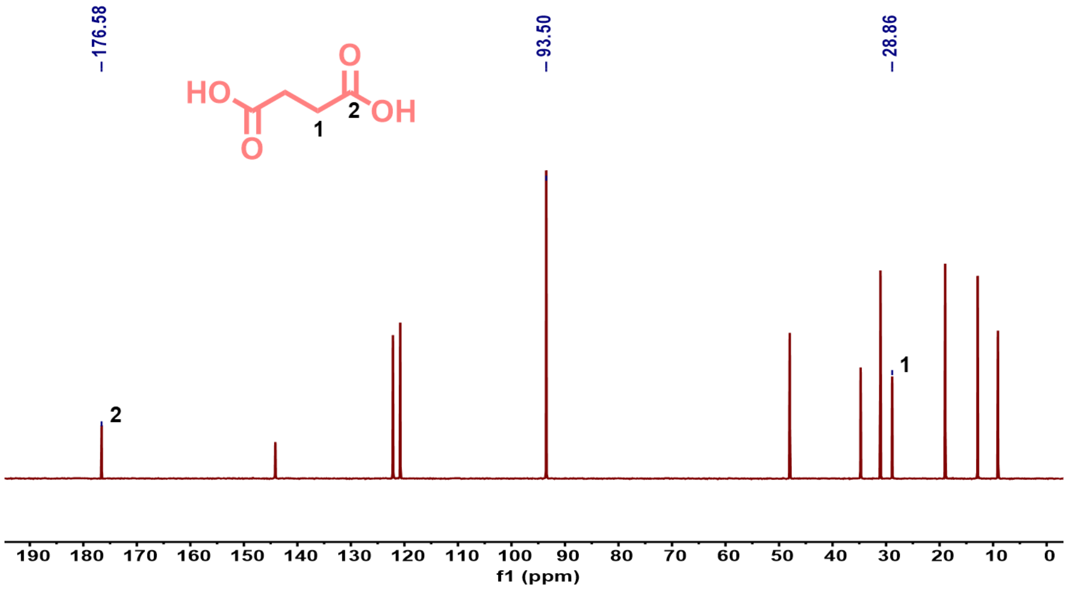


**Supplementary Figure 28. NMR spectra of commercial succinic acid in [BMMIm]Br.** (**a**) $^{1}H$ NMR spectrum (1,3,5-trioxane as the internal standard, 5.30 ppm); (**b**) $^{13}C$ NMR spectrum (1,3,5- trioxane as the internal standard, 93.50 ppm).

(a)

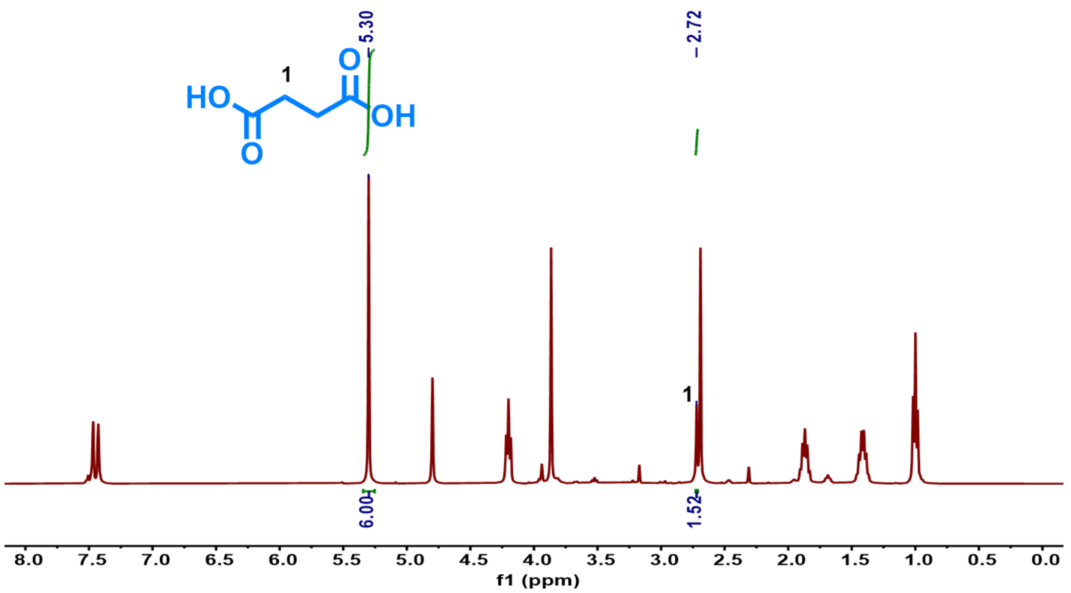


(b)

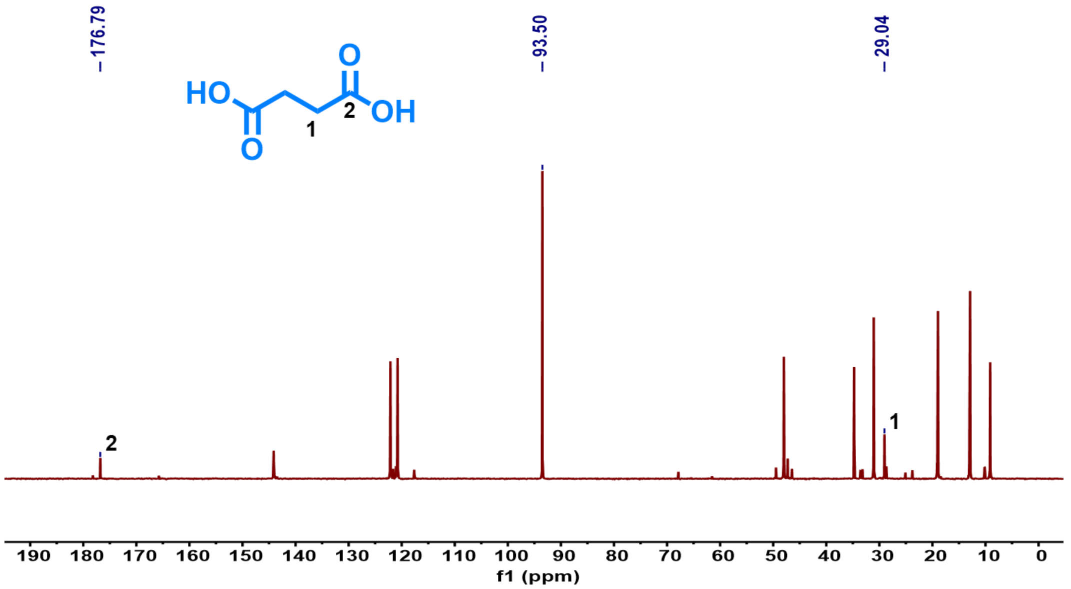


**Supplementary Figure 29. NMR spectra of the liquid reaction solution from PBS powder decomposition over [BMMIm]Br-Pd/C under the $H_2$ atmosphere.** (**a**) $^{1}H$ NMR spectrum (1,3,5-trioxane as the internal standard, 5.30 ppm); (**b**) $^{13}C$ NMR spectrum (1,3,5- trioxane as the internal standard, 93.50 ppm).

(a)

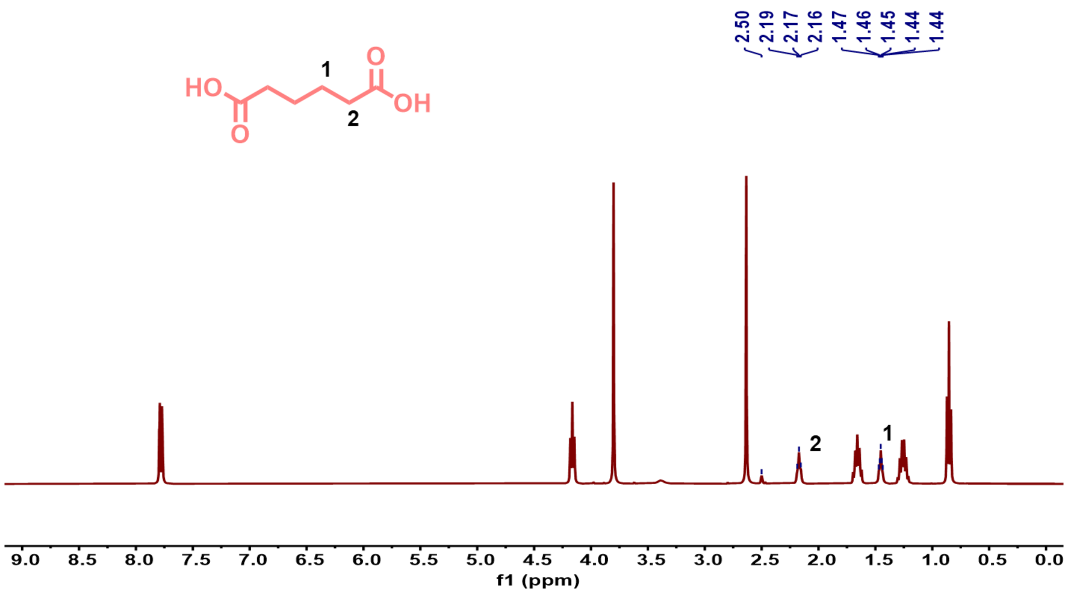


(b)

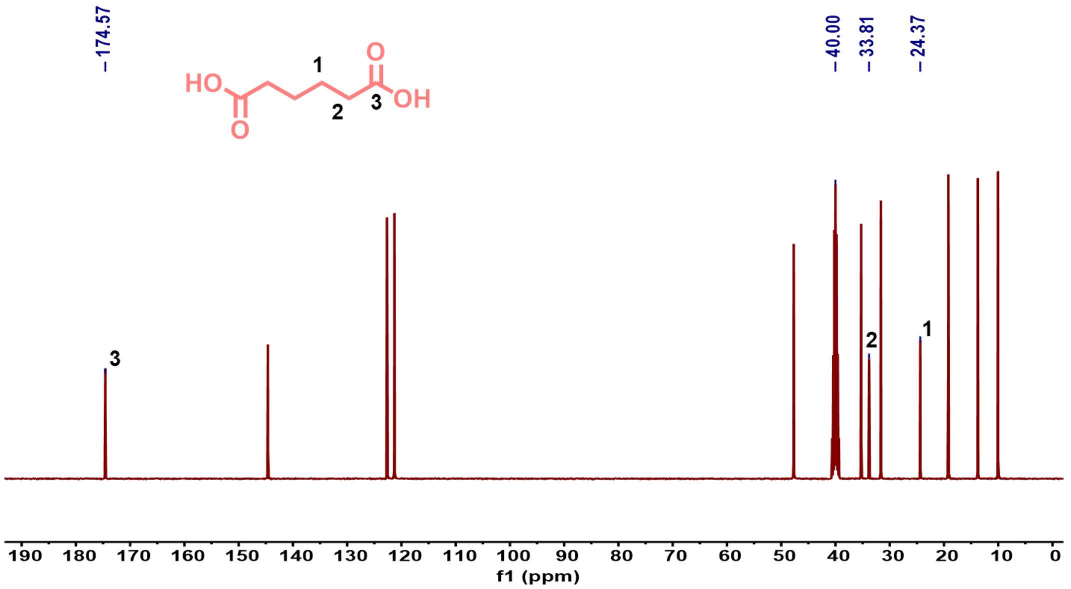


**Supplementary Figure 30. NMR spectra of commercial adipic acid in [BMMIm]Br.** (**a**) $^{1}H$ NMR spectrum (DMSO-d6, 2.50 ppm); (**b**) $^{13}C$ NMR spectrum (DMSO-d6, 40.00 ppm).

(a)

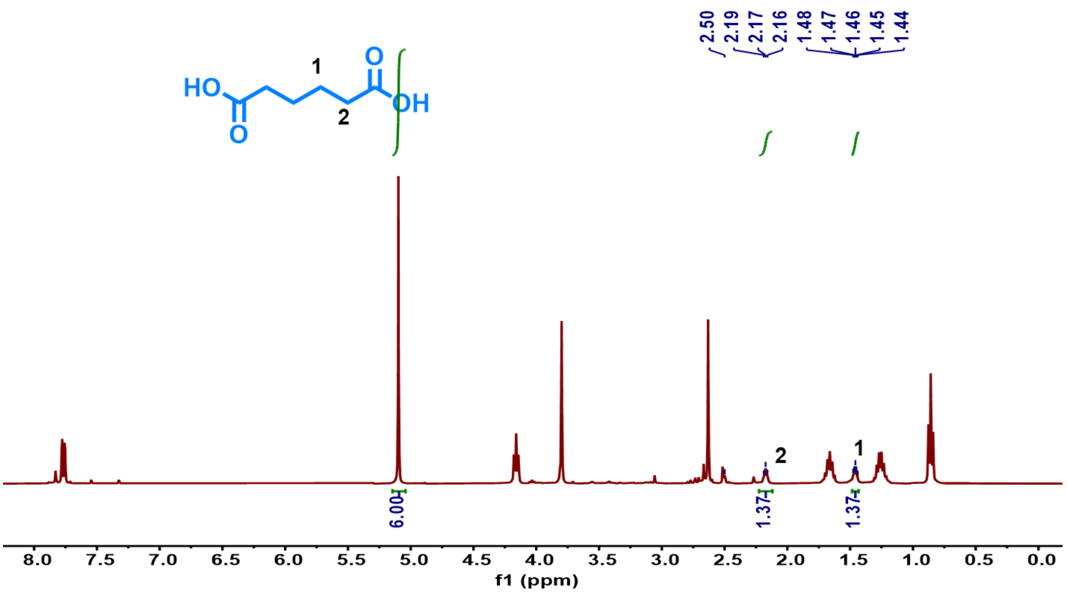


(b)

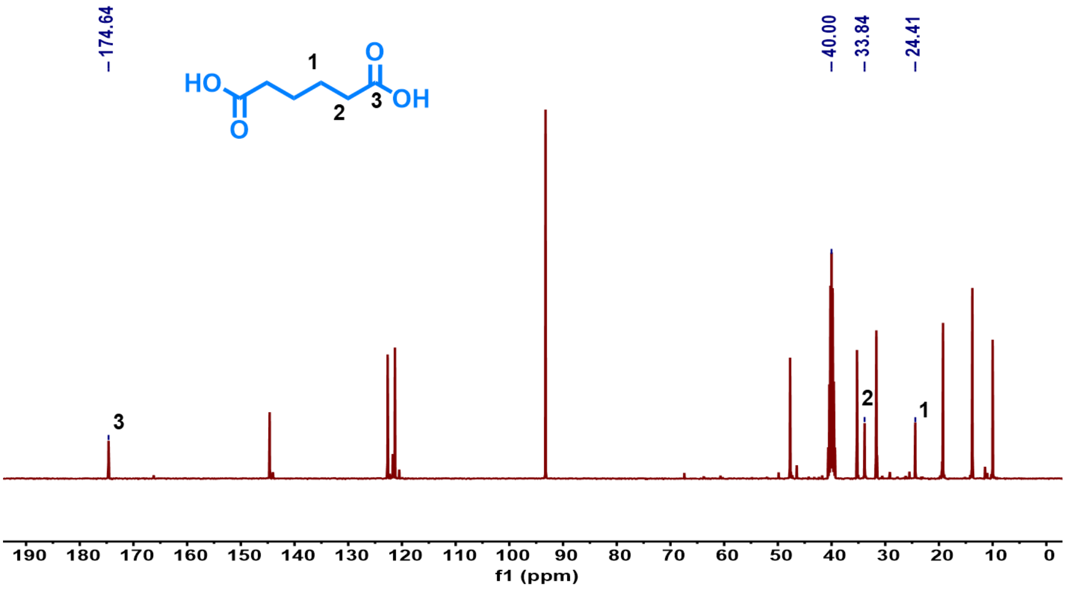


**Supplementary Figure 31. NMR spectra of the liquid reaction solution from PBA granule decomposition over [BMMIm]Br-Pd/C under the $H_2$ atmosphere.** (**a**) $^{1}H$ NMR spectrum (1,3,5-trioxane as the internal standard, DMSO-d6, 2.50 ppm); (**b**) $^{13}C$ NMR spectrum (1,3,5-trioxane as the internal standard, DMSO-d6, 40.00 ppm).

(a)

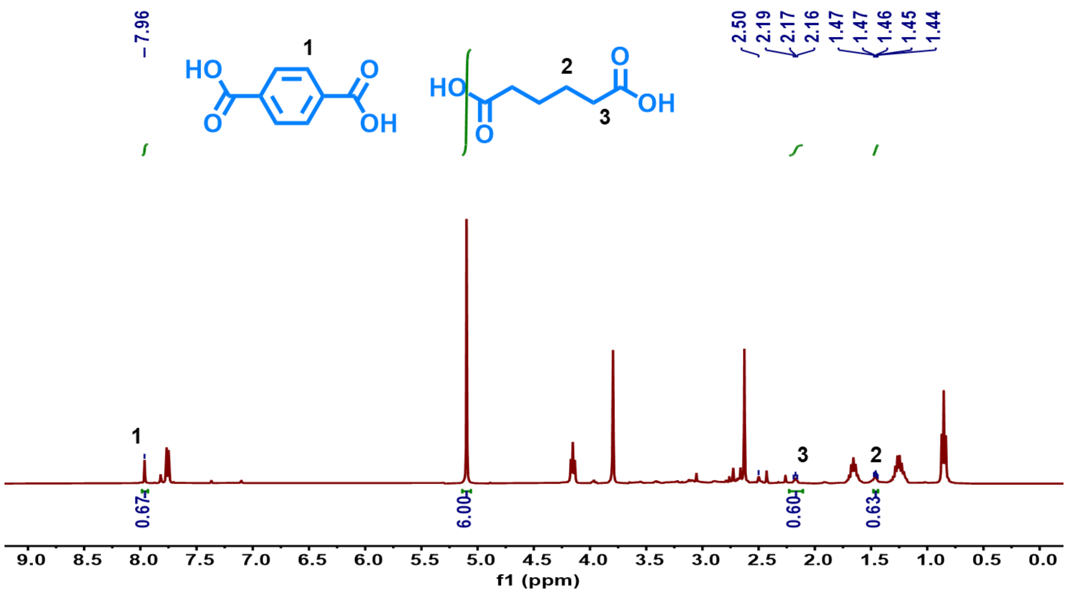


(b)

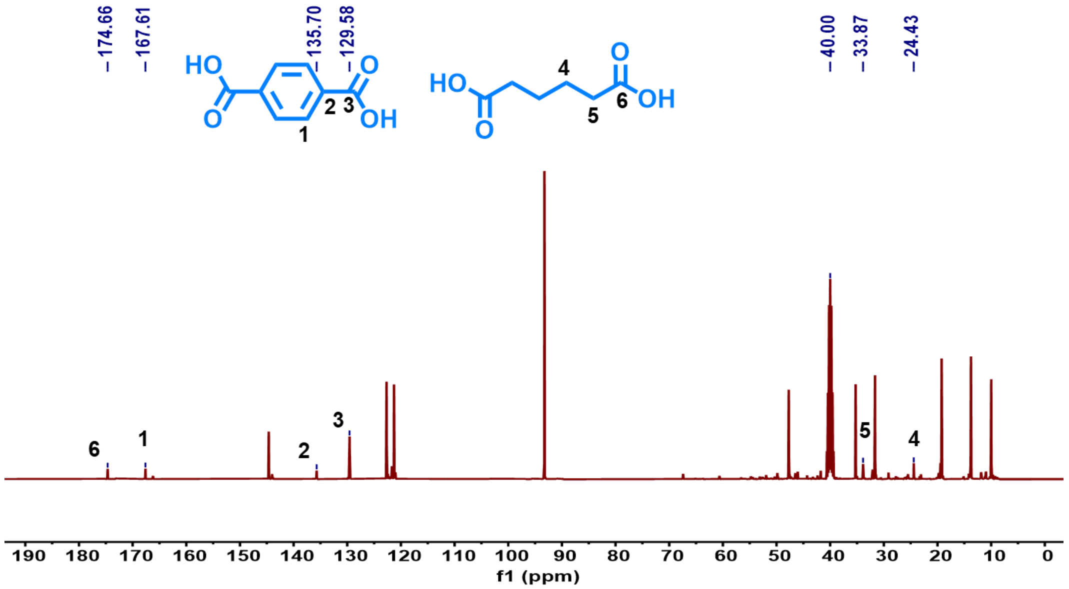


**Supplementary Figure 32. NMR spectra of the liquid reaction solution from PBAT granule decomposition over [BMMIm]Br-Pd/C under the $H_2$ atmosphere.** (**a**) $^{1}H$ NMR spectrum (1,3,5-trioxane as the internal standard, DMSO-d6, 2.50 ppm); (**b**) $^{13}C$ NMR spectrum (1,3,5-trioxane as the internal standard, DMSO-d6, 40.00 ppm).

(a)

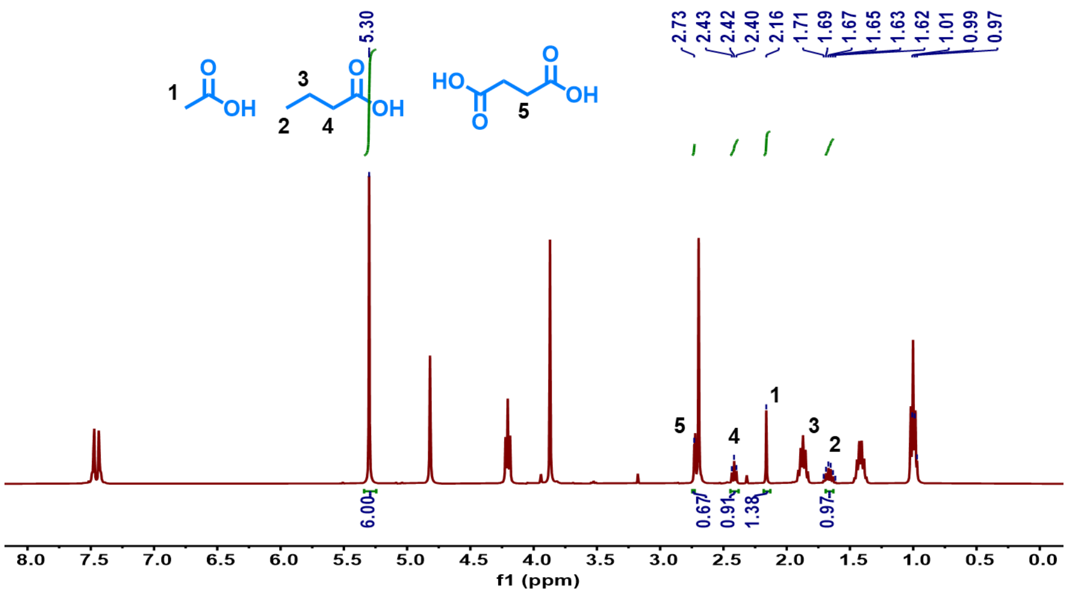


(b)

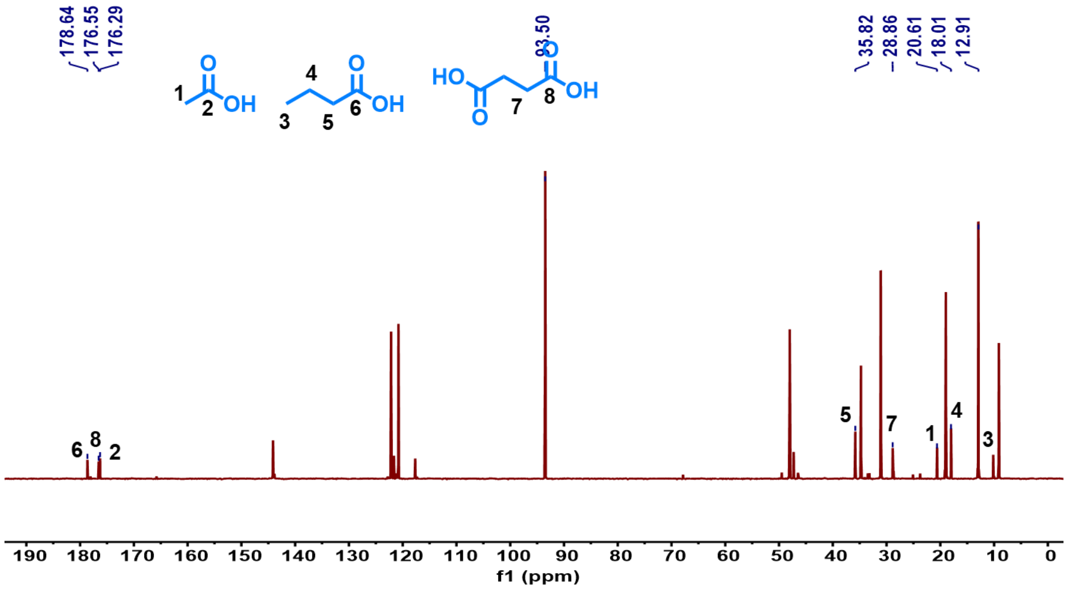


**Supplementary Figure 33. NMR spectra of the liquid reaction solution from the decomposition for the mixture of PGA, PHB and PBS over [BMMIm]Br-Pd/C under the $H_2$ atmosphere.** (**a**) $^1$H NMR spectrum (1,3,5-trioxane as the internal standard, 5.30 ppm); (**b**) $^{13}$C NMR spectrum (1,3,5- trioxane as the internal standard, 93.50 ppm).

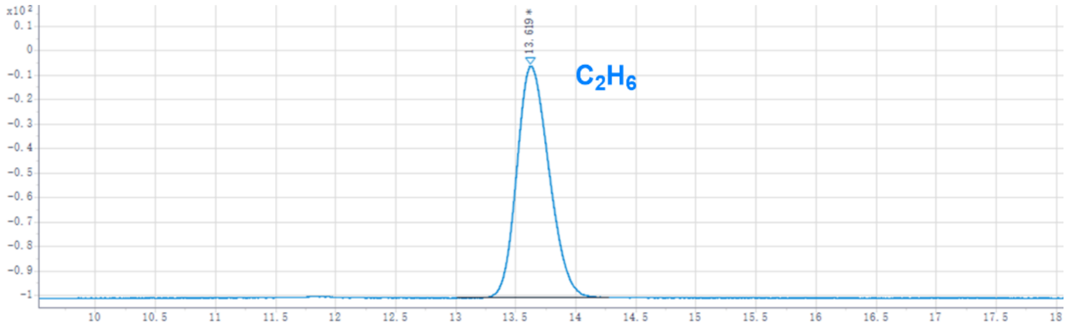


**Supplementary Figure 34. GC spectrum of the gaseous products from PET powder decomposition over [BMMIm]Br-Pd/C under the $H_2$ atmosphere.**

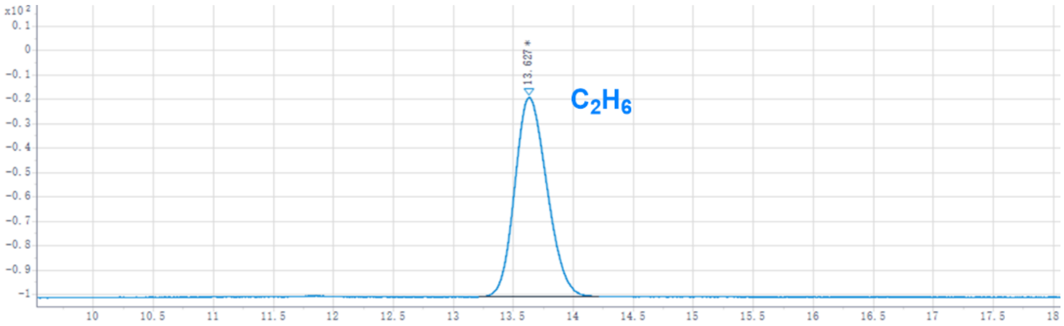


**Supplementary Figure 35. GC spectrum of the gaseous products from the decomposition of PET piece from plastic bottle over [BMMIm]Br-Pd/C under the $H_2$ atmosphere.**

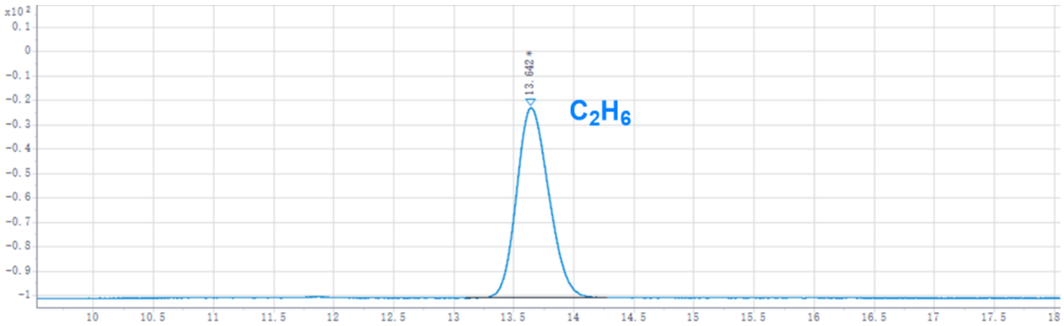


**Supplementary Figure 36. GC spectrum of the gaseous products from the decomposition of PET piece from plastic bottle over [BMMIm]Br-Pd/C in toluene under the $H_2$ atmosphere.**

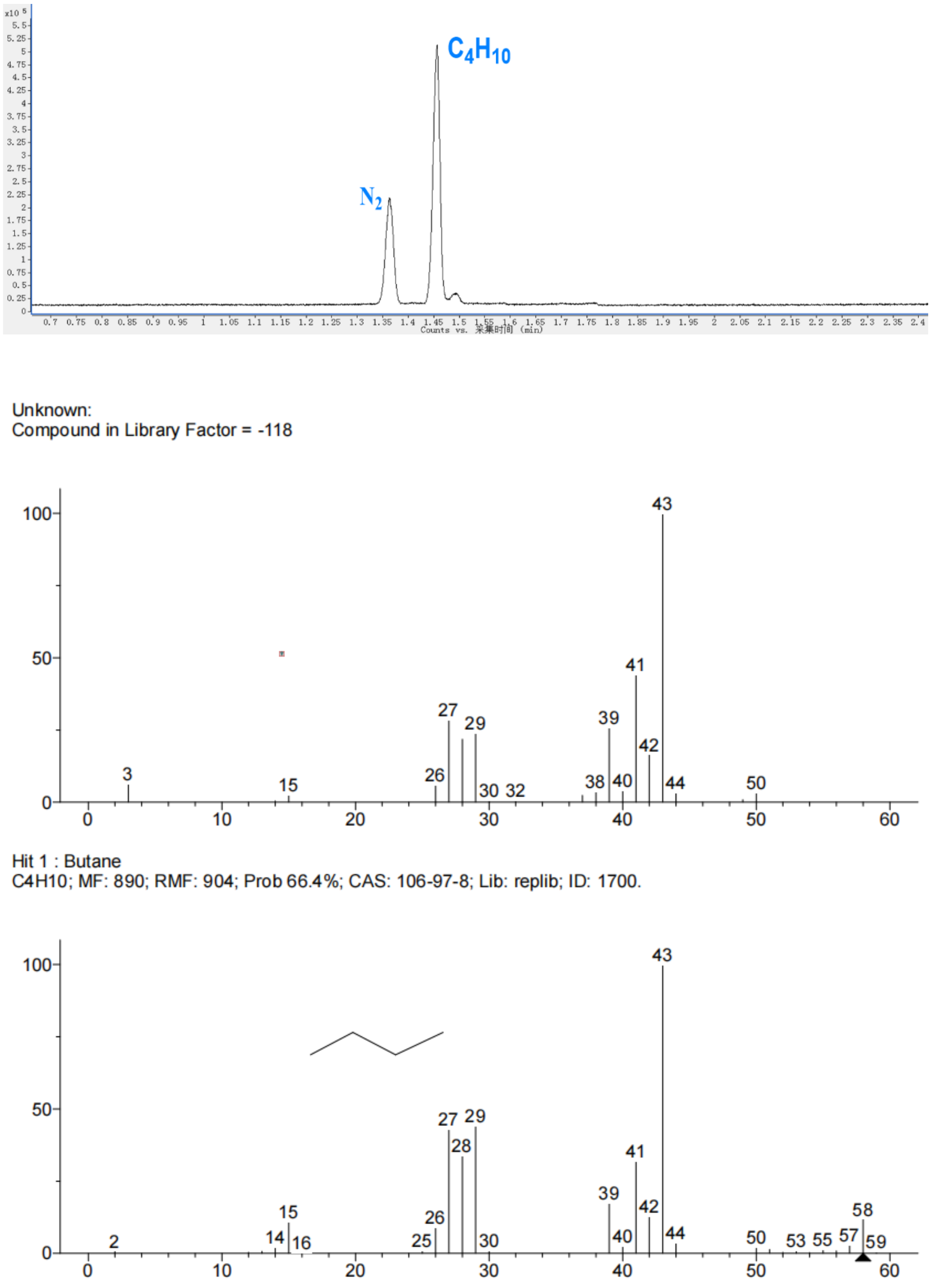


**Supplementary Figure 37. GC-MS spectrum of the gaseous products from PBT powder decomposition over [BMMIm]Br-Pd/C under the $H_2$ atmosphere.**

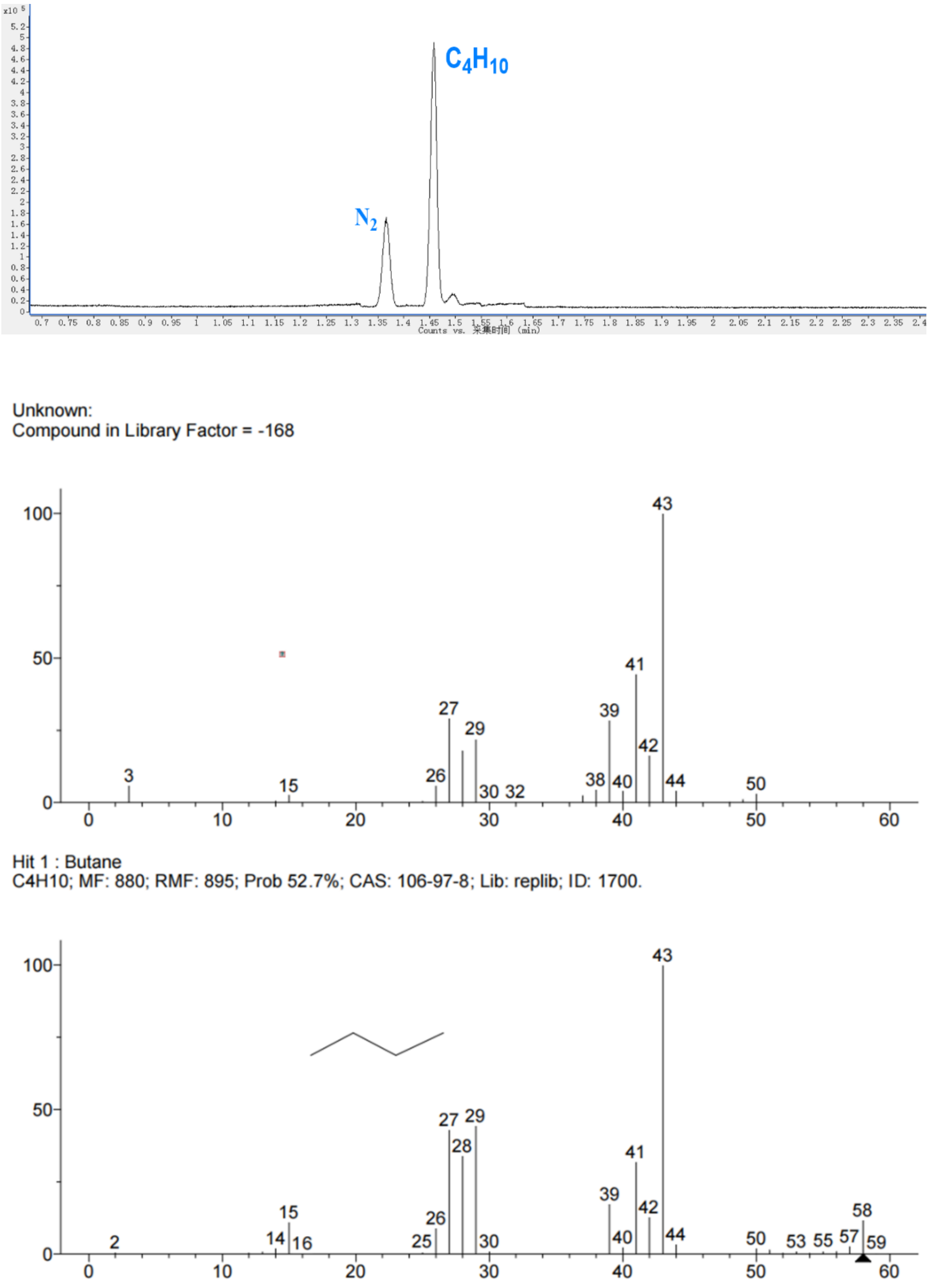


**Supplementary Figure 38. GC-MS spectrum of the gaseous products from PBS powder decomposition over [BMMIm]Br-Pd/C under the $H_2$ atmosphere.**

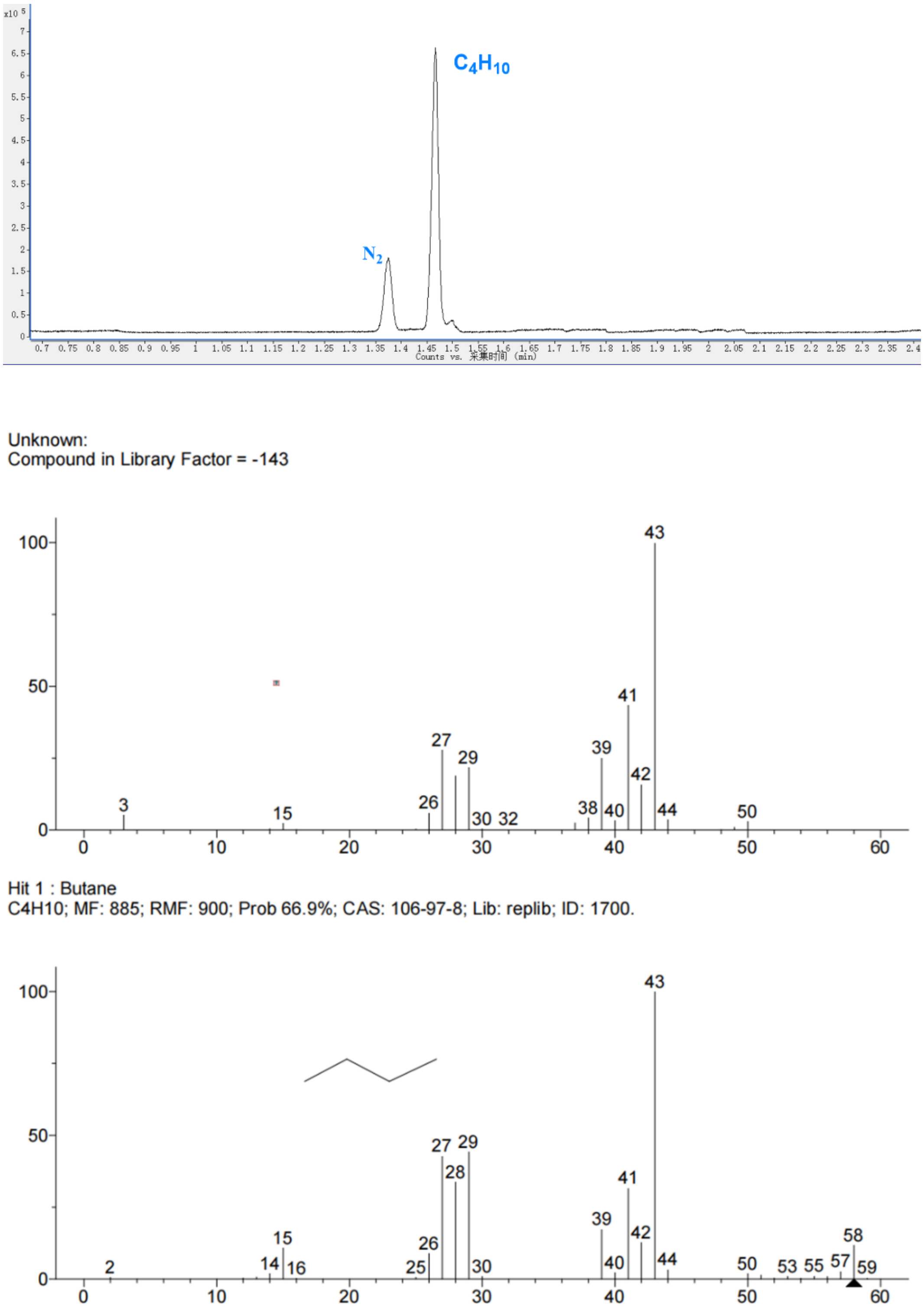


**Supplementary Figure 39. GC-MS spectrum of the gaseous products from PBA granule decomposition over [BMMIm]Br-Pd/C under the $H_2$ atmosphere.**

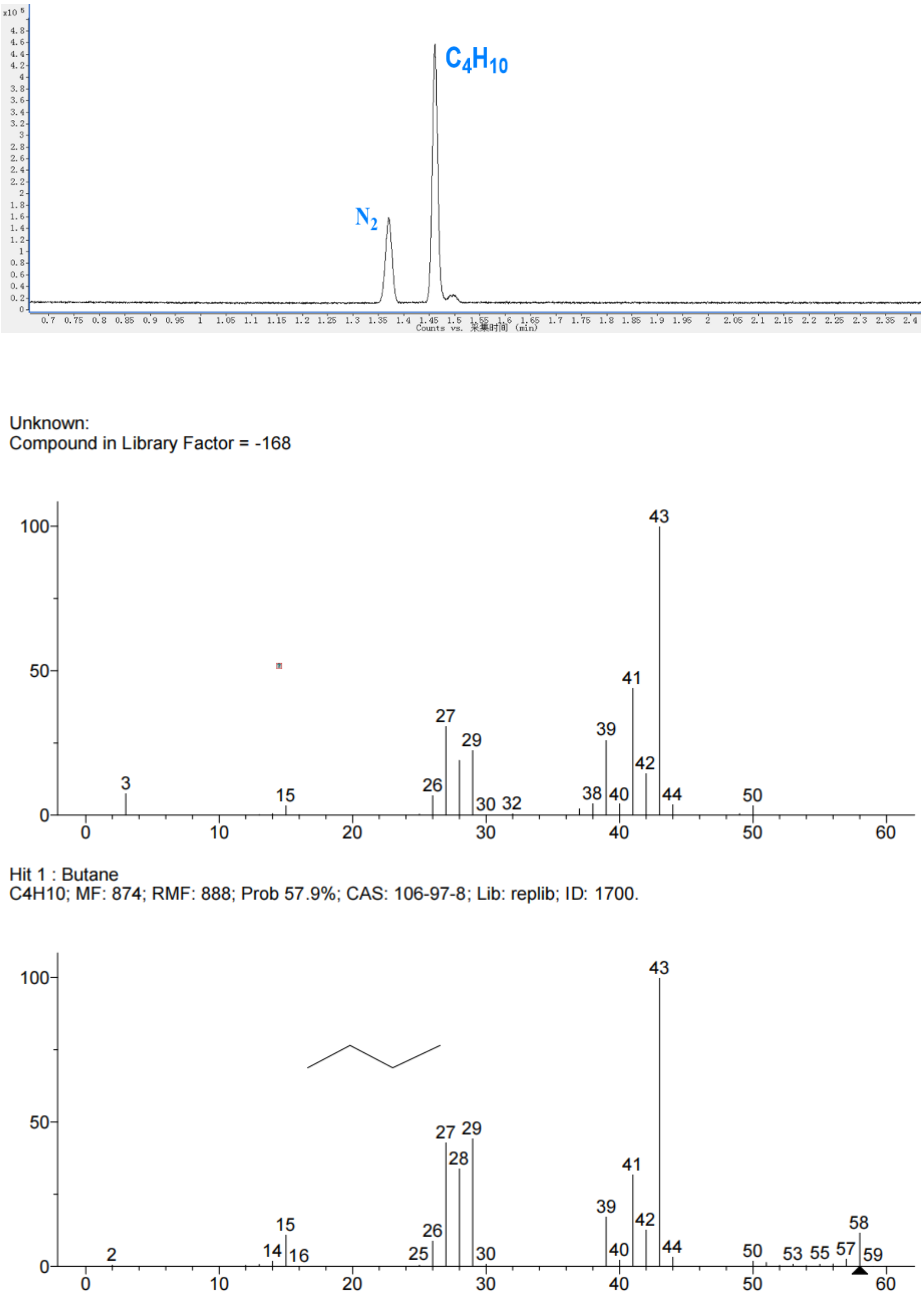


**Supplementary Figure 40. GC-MS spectrum of the gaseous products from PBAT granule decomposition over [BMMIm]Br-Pd/C under the $H_2$ atmosphere.**

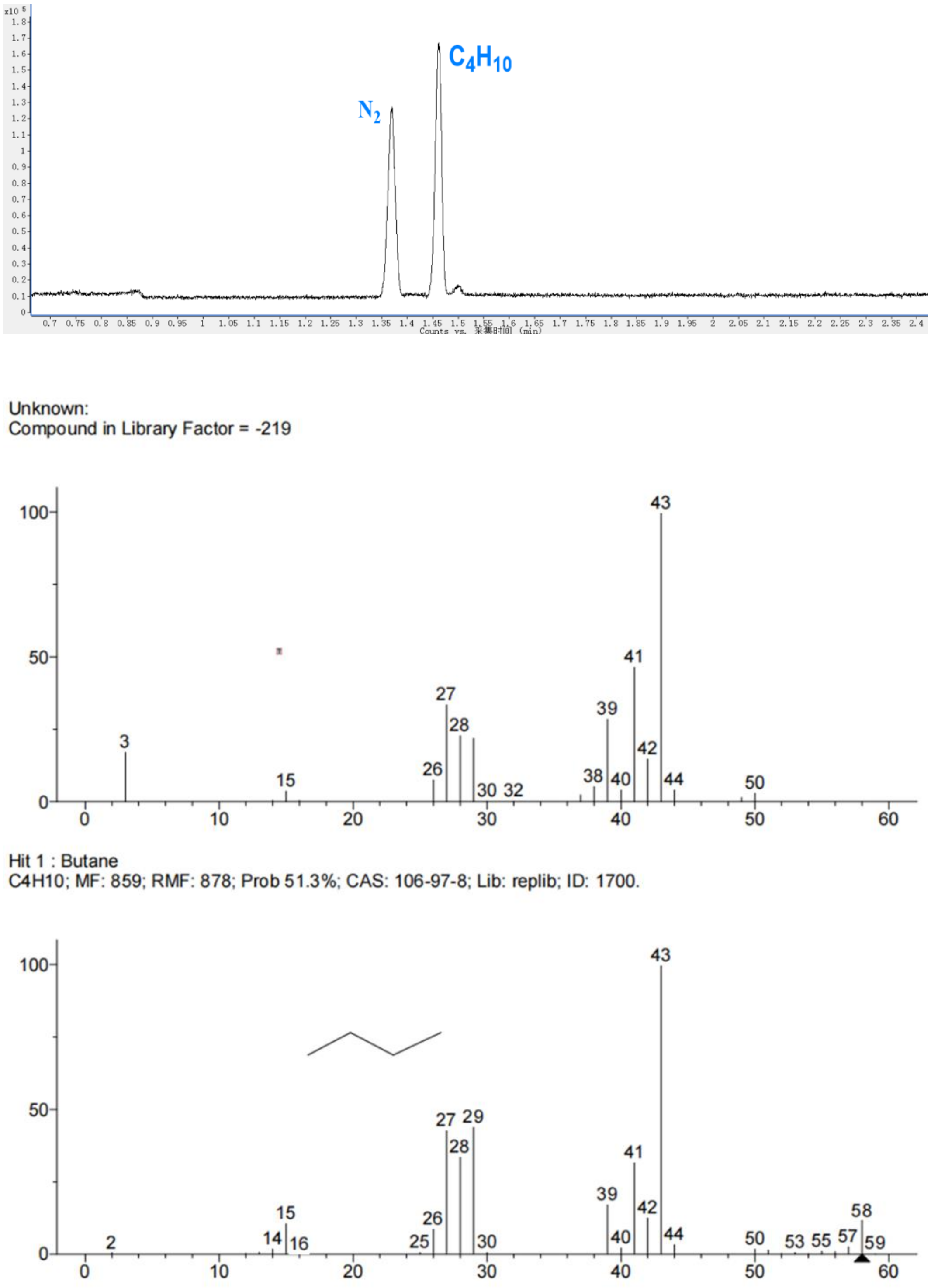


**Supplementary Figure 41. GC-MS spectrum of the gaseous products from the decomposition for the mixture of PGA, PHB and PBS over [BMMIm]Br-Pd/C under the $H_2$ atmosphere.**

(a)

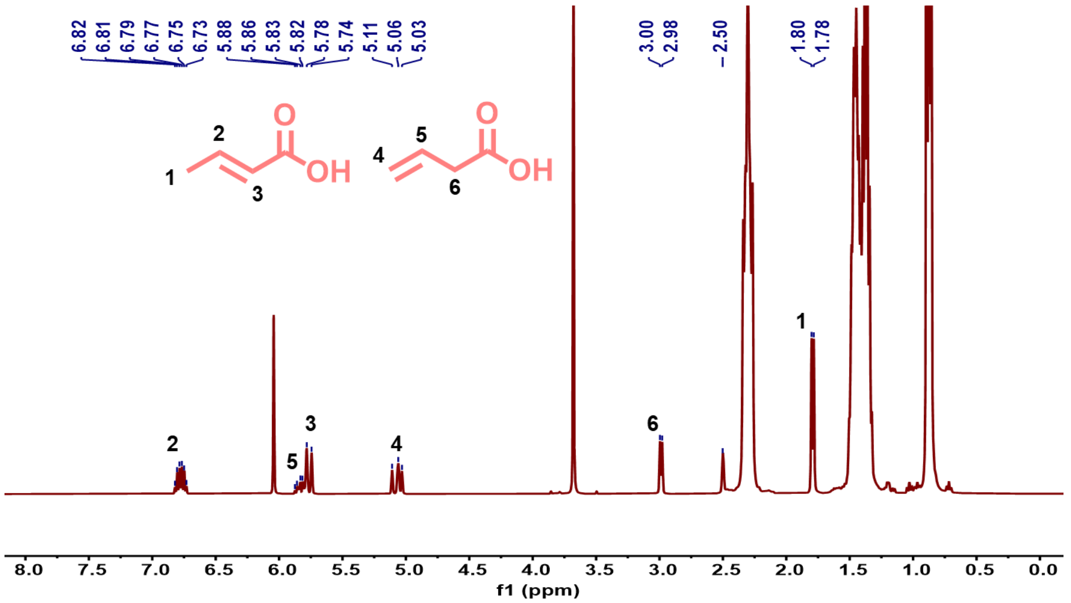


(b)

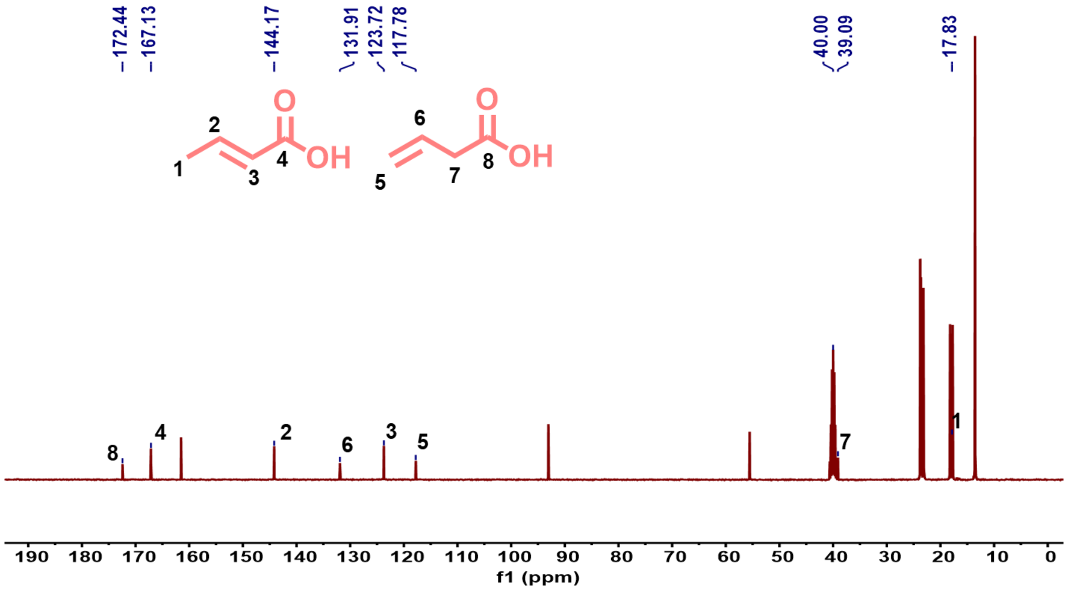


**Supplementary Figure 42. NMR spectra of commercial crotonic acid and 3-butenoic acid in [P4444]Br.** (**a**) $^{1}H$ NMR spectrum (1,3,5-trimethoxybenzene as the internal standard, DMSO-d6, 2.50 ppm); (**b**) $^{13}C$ NMR spectrum (1,3,5-trimethoxybenzene as the internal standard, DMSO-d6, 40.00 ppm).

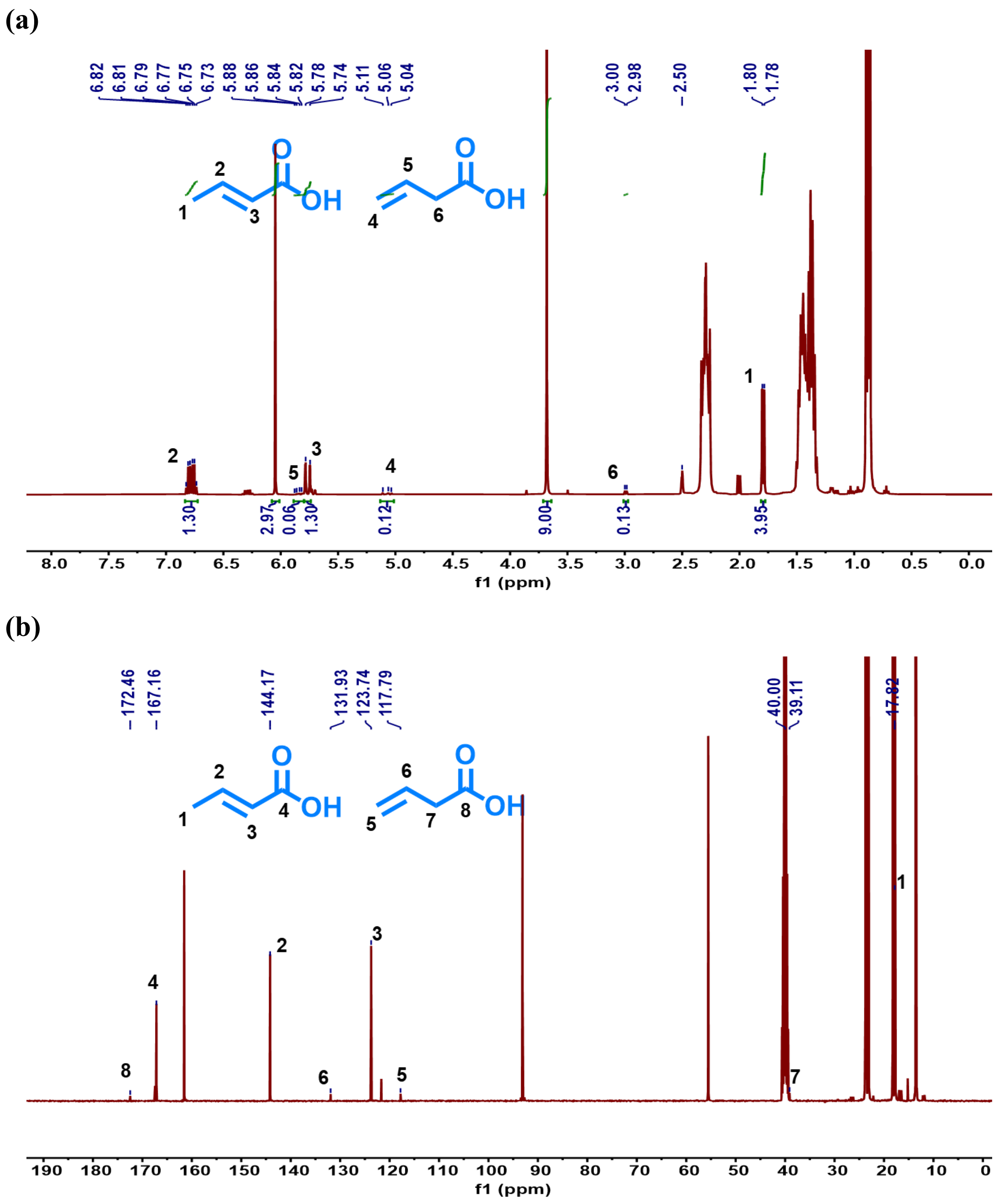


**Supplementary Figure 43. NMR spectra of the liquid reaction solution from PHB powder deconstruction over [$P_{4444}$]Br under the $N_2$ atmosphere.** (**a**) $^1$H NMR spectrum (1,3,5-trimethoxybenzene as the internal standard, DMSO-d6, 2.50 ppm); (**b**) $^{13}$C NMR spectrum (1,3,5-trimethoxybenzene as the internal standard, DMSO-d6, 40.00 ppm).

(a)

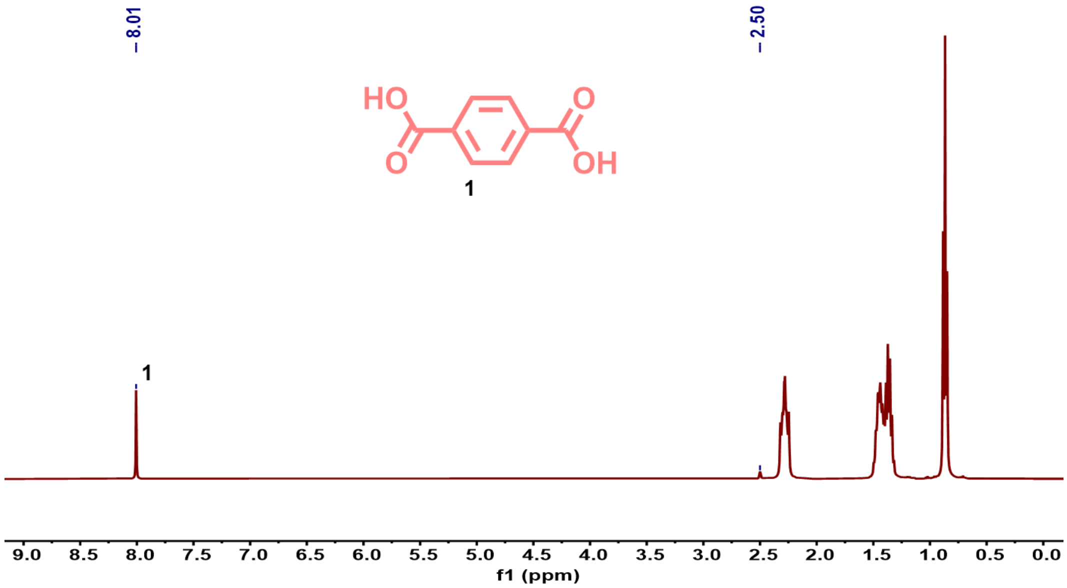


(b)

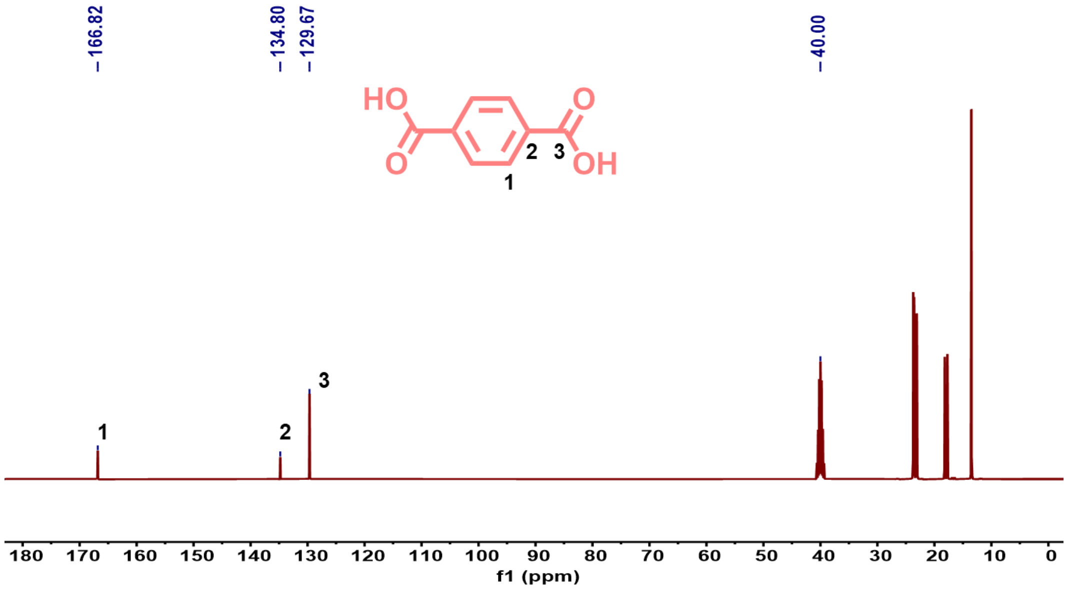


**Supplementary Figure 44. NMR spectra of commercial terephthalic acid in [$P_{4444}$]Br.** (**a**) $^{1}$H NMR spectrum (DMSO-d6, 2.50 ppm); (**b**) $^{13}$C NMR spectrum (DMSO-d6, 40.00 ppm).

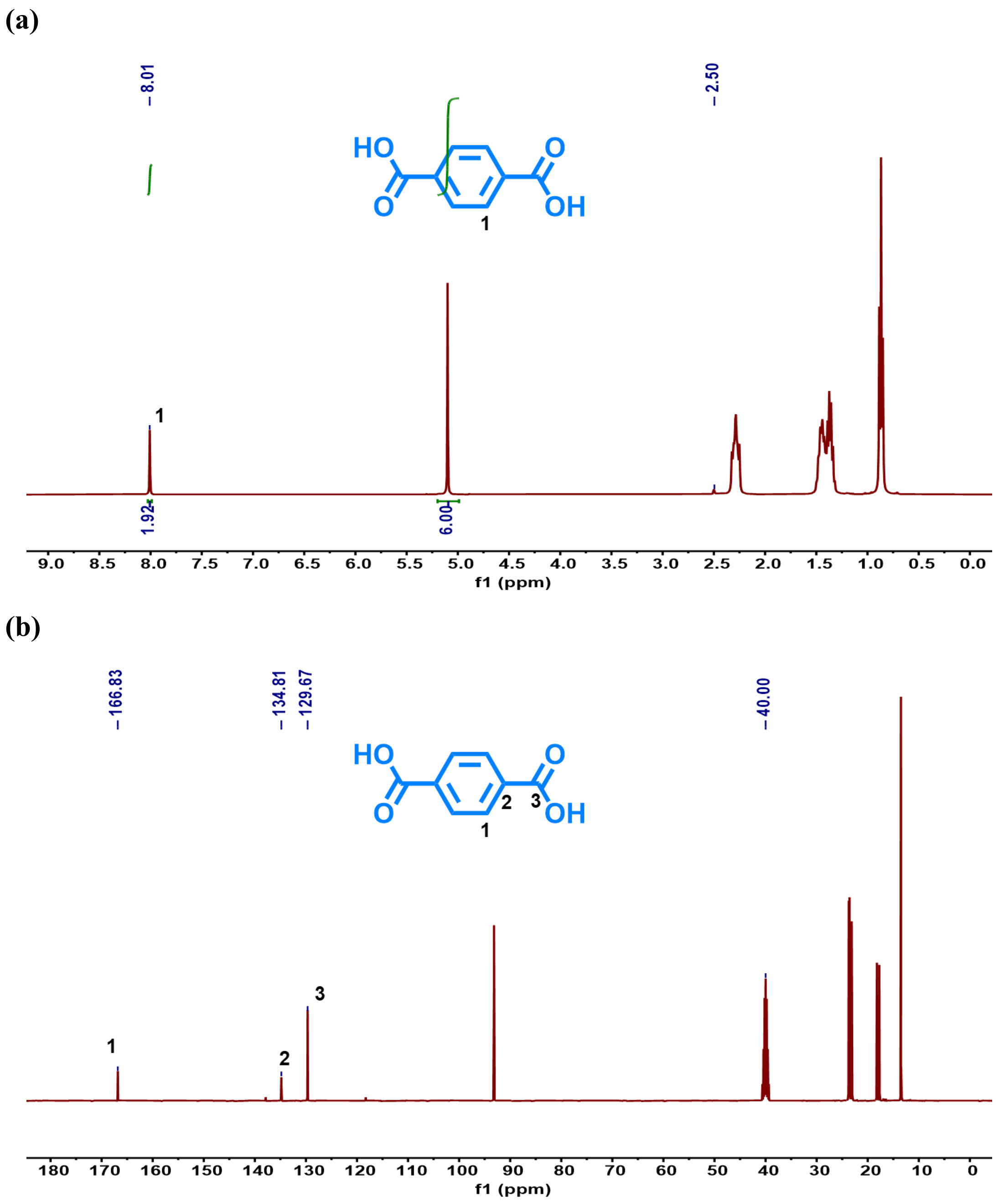


**Supplementary Figure 45. NMR spectra of the liquid reaction solution from PBT powder deconstruction over [$P_{4444}$]Br under the $N_2$ atmosphere.** (**a**) $^{1}H$ NMR spectrum (1,3,5-trioxane as the internal standard, DMSO-d6, 2.50 ppm); (**b**) $^{13}C$ NMR spectrum (1,3,5-trioxane as the internal standard, DMSO-d6, 40.00 ppm).

(a)

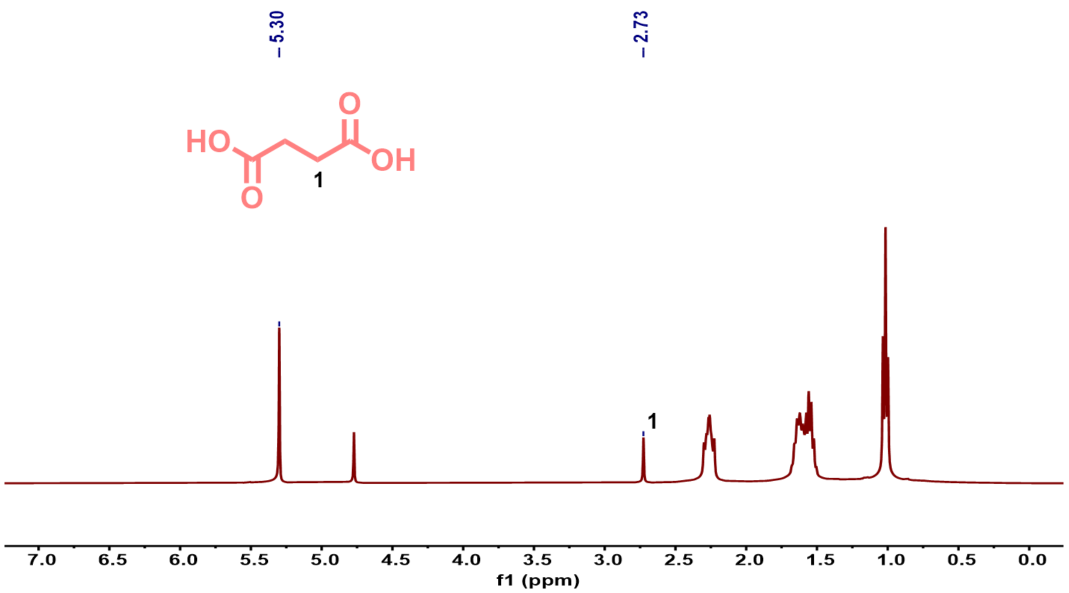


(b)

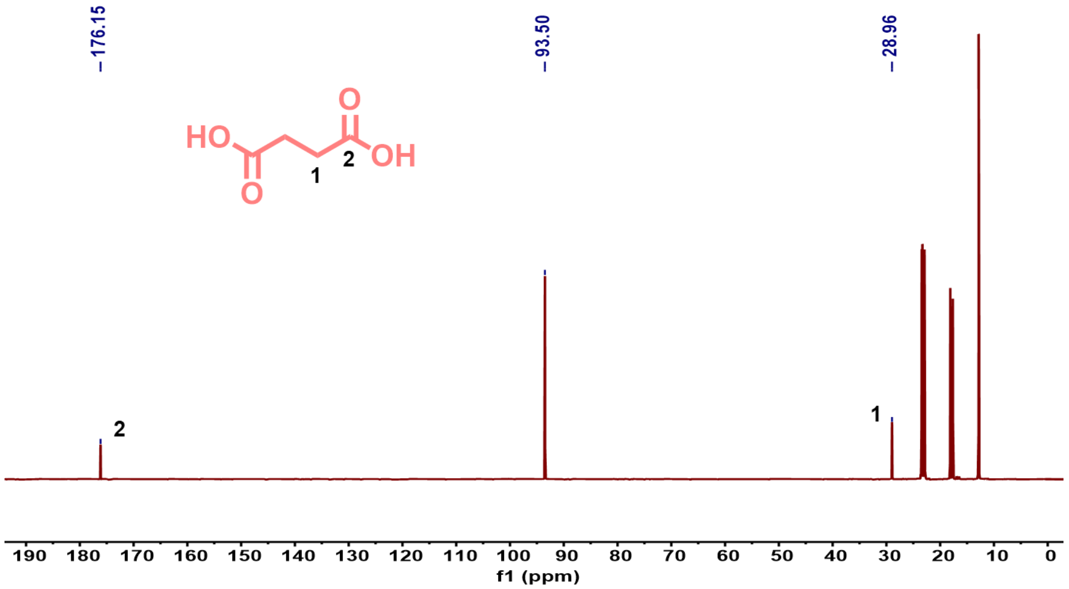


**Supplementary Figure 46. NMR spectra of commercial succinic acid in [$P_{4444}$]Br.** (**a**) $^{1}$H NMR spectrum (1,3,5-trioxane as the internal standard, 5.30 ppm); (**b**) $^{13}$C NMR spectrum (1,3,5- trioxane as the internal standard, 93.50 ppm).

(a)

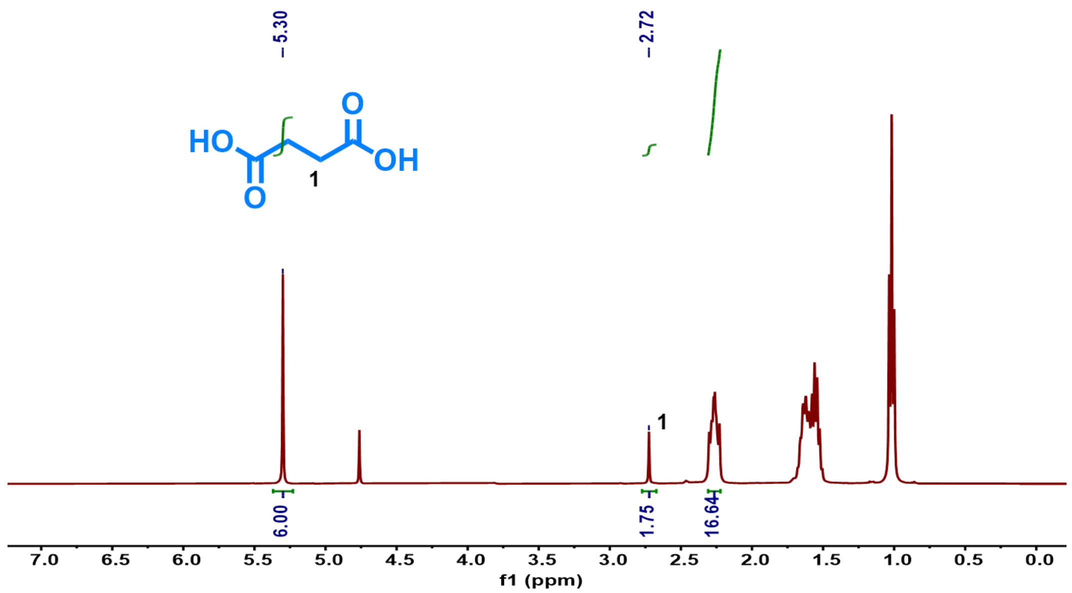


(b)

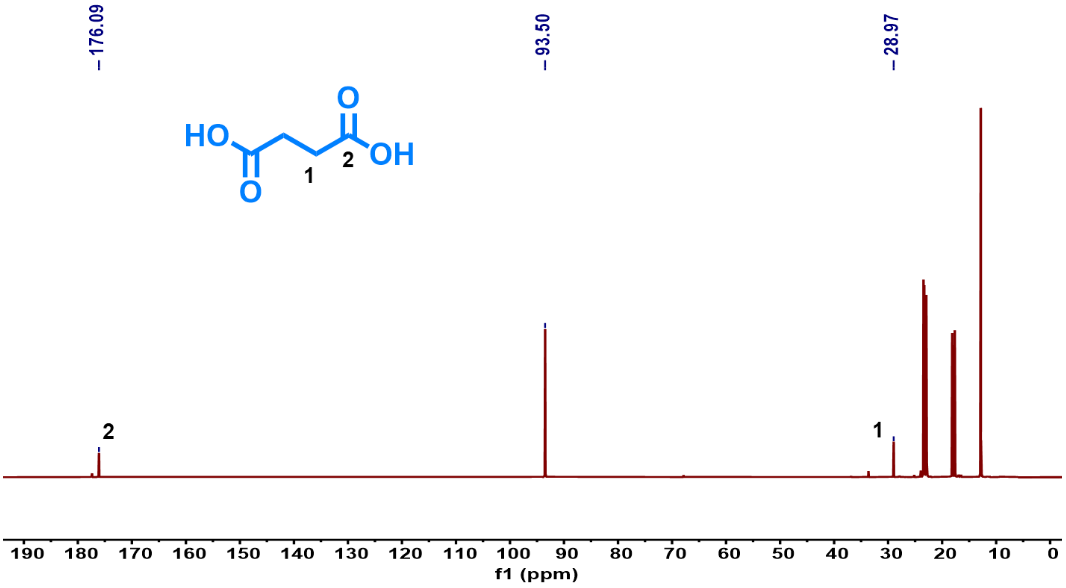


**Supplementary Figure 47. NMR spectra of the liquid reaction solution from PBS powder deconstruction over [$P_{4444}$]Br under the $N_2$ atmosphere.** (**a**) $^{1}H$ NMR spectrum (1,3,5-trioxane as the internal standard, 5.30 ppm); (**b**) $^{13}C$ NMR spectrum (1,3,5- trioxane as the internal standard, 93.50 ppm).

(a)

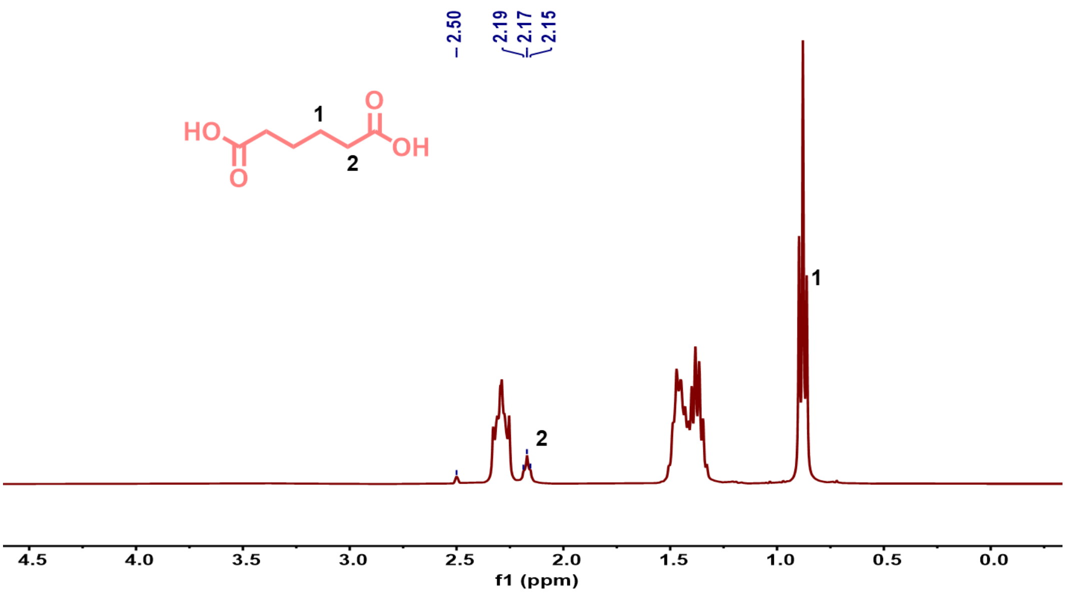


(b)

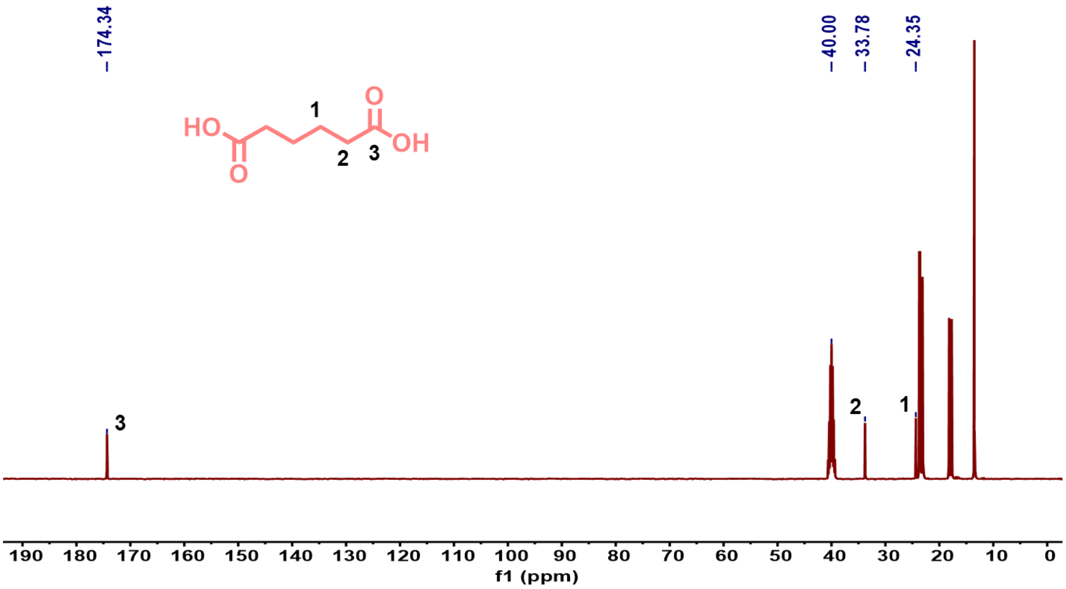


**Supplementary Figure 48. NMR spectra of commercial adipic acid in [P$_{4444}$]Br.** (**a**) $^{1}$H NMR spectrum (DMSO-d6, 2.50 ppm); (**b**) $^{13}$C NMR spectrum (DMSO-d6, 40.00 ppm).

(a)

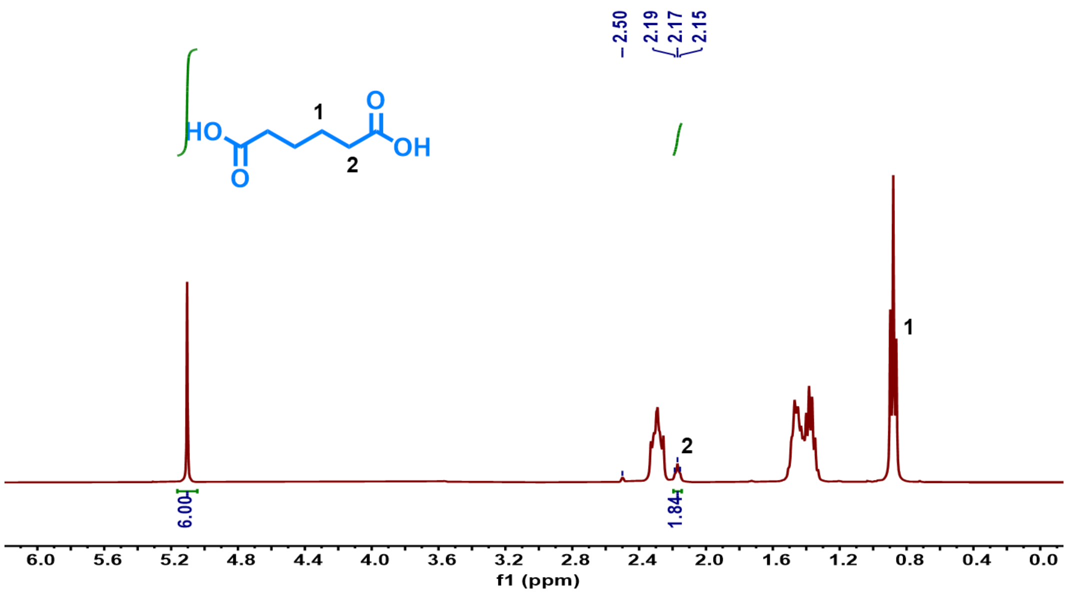


(b)

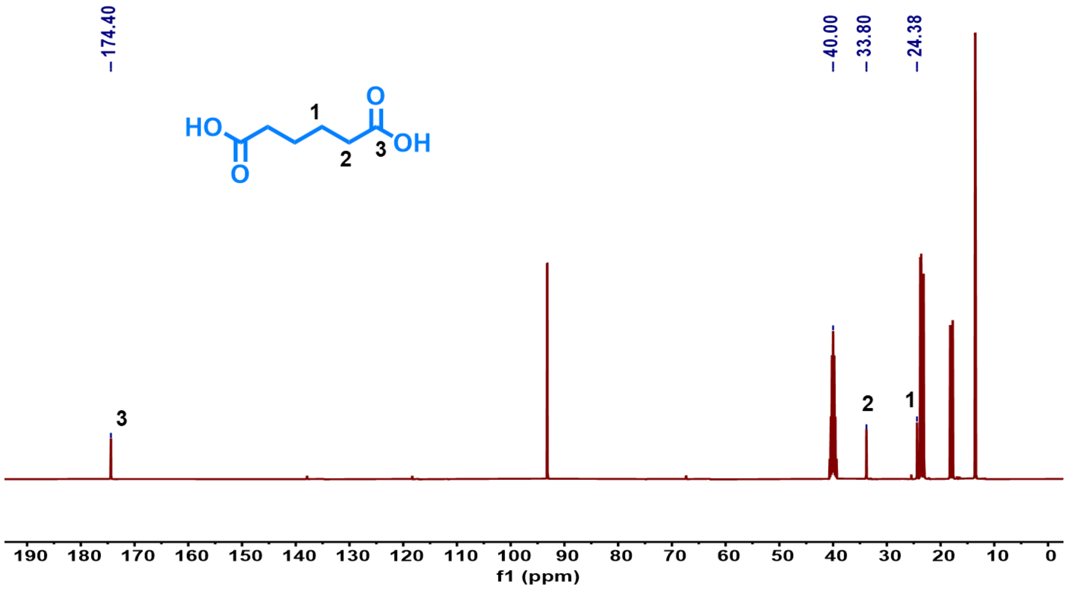


**Supplementary Figure 49. NMR spectra of the liquid reaction solution from PBA granule deconstruction over [P4444]Br under the $N_2$ atmosphere.** (**a**) $^1$H NMR spectrum (1,3,5-trioxane as the internal standard, DMSO-d6, 2.50 ppm); (**b**) $^{13}$C NMR spectrum (1,3,5-trioxane as the internal standard, DMSO-d6, 40.00 ppm).

(a)

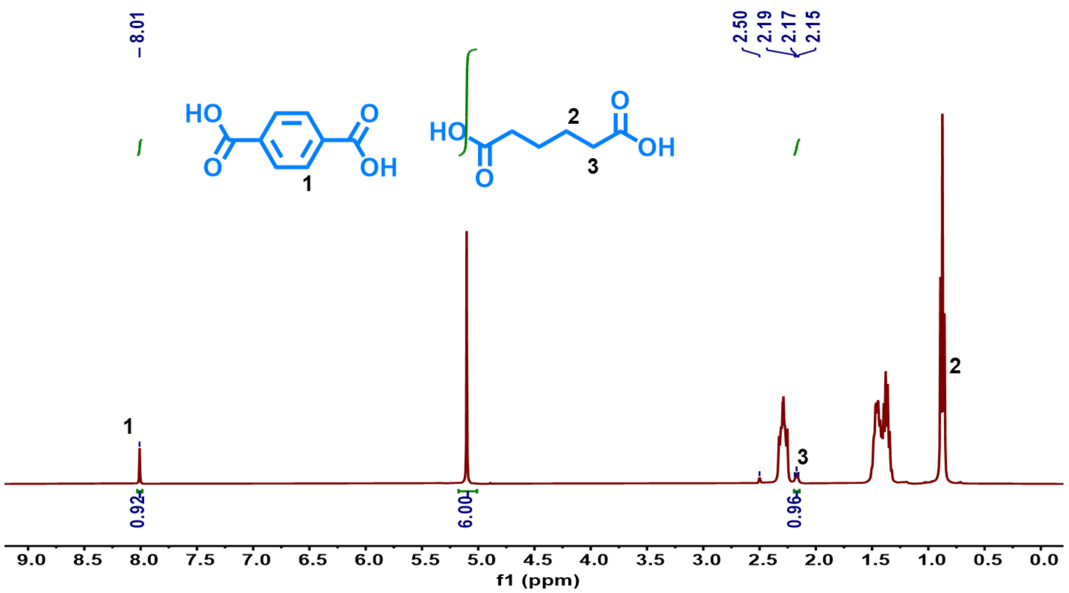


(b)

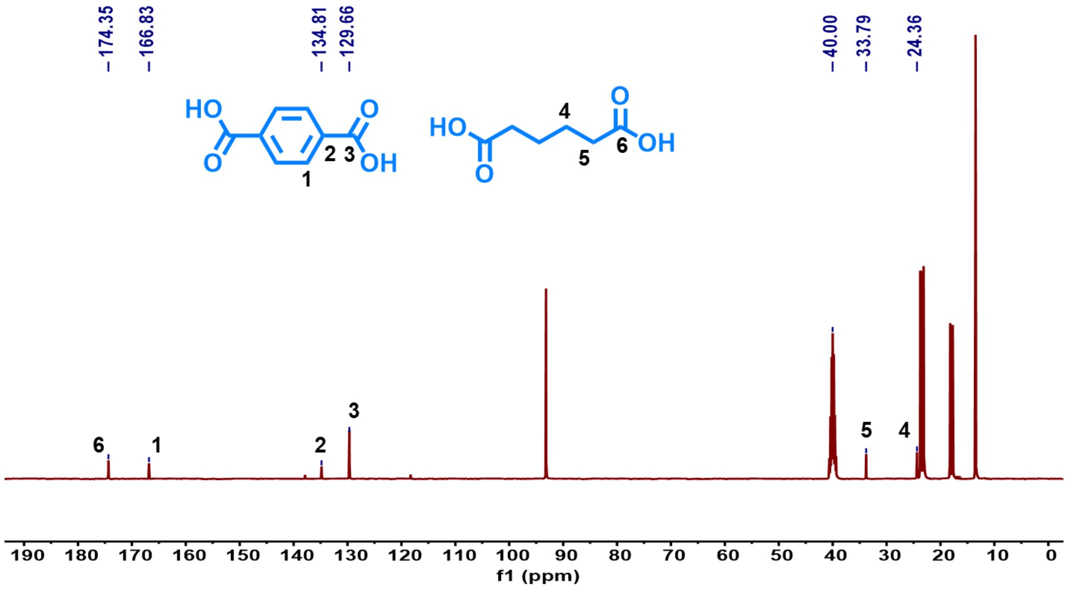


**Supplementary Figure 50. NMR spectra of the liquid reaction solution from PBAT granule deconstruction over $[P_{4444}]Br$ under the $N_2$ atmosphere.** (**a**) $^1H$ NMR spectrum (1,3,5-trioxane as the internal standard, DMSO-d6, 2.50 ppm); (**b**) $^{13}C$ NMR spectrum (1,3,5-trioxane as the internal standard, DMSO-d6, 40.00 ppm).

(a)

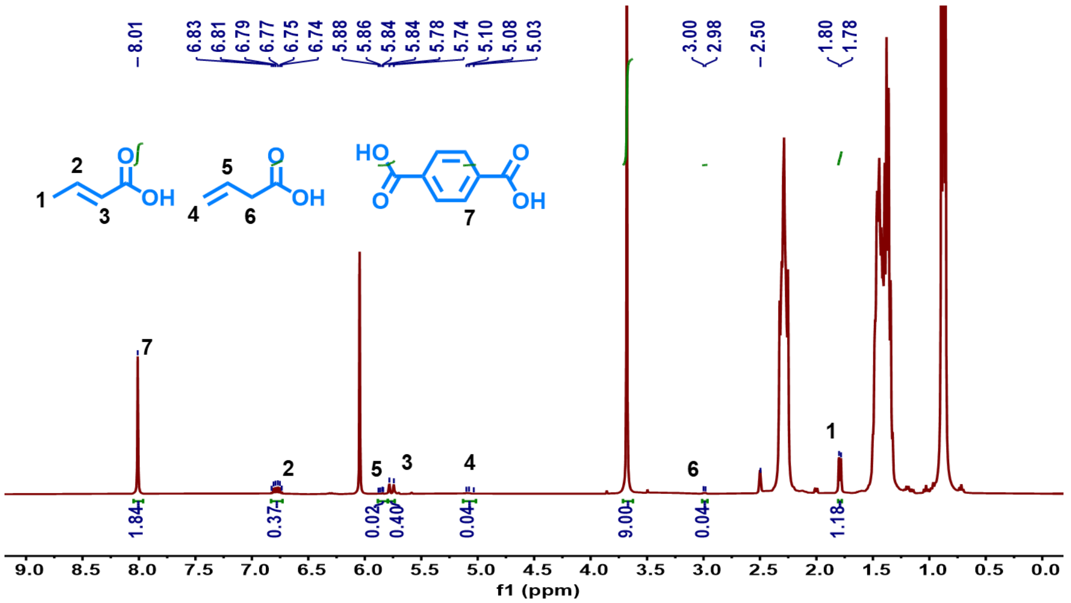


(b)

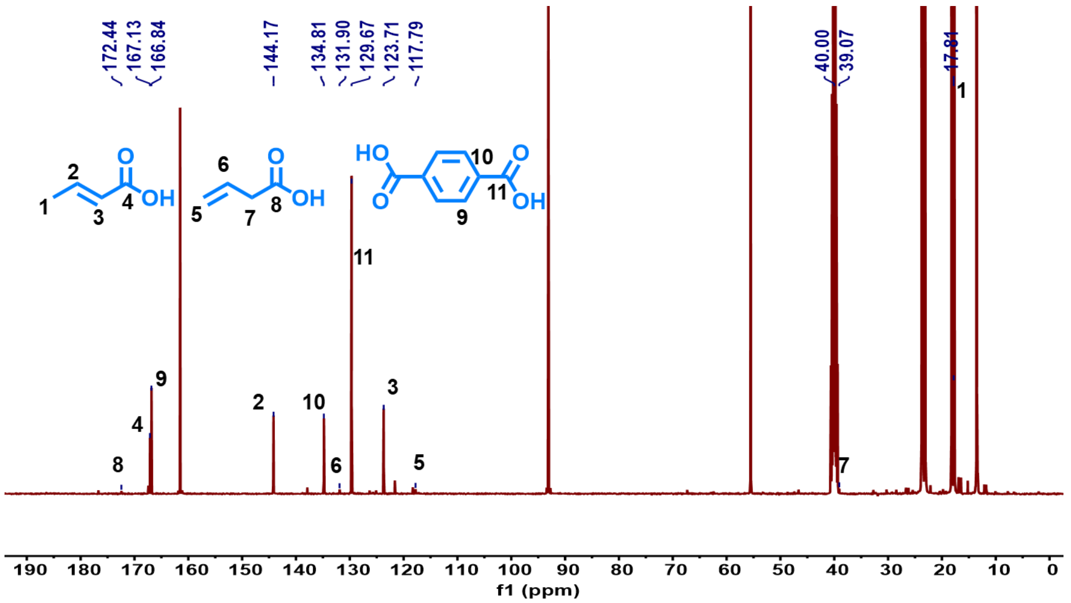


**Supplementary Figure 51. NMR spectra of the liquid reaction solution from the deconstruction for the mixture of PHB and PBT powder over [$P_{4444}$]Br under the $N_2$ atmosphere.** (**a**) $^{1}H$ NMR spectrum (1,3,5-trimethoxybenzene as the internal standard, DMSO-d6, 2.50 ppm); (**b**) $^{13}C$ NMR spectrum (1,3,5-trimethoxybenzene as the internal standard, DMSO-d6, 40.00 ppm).

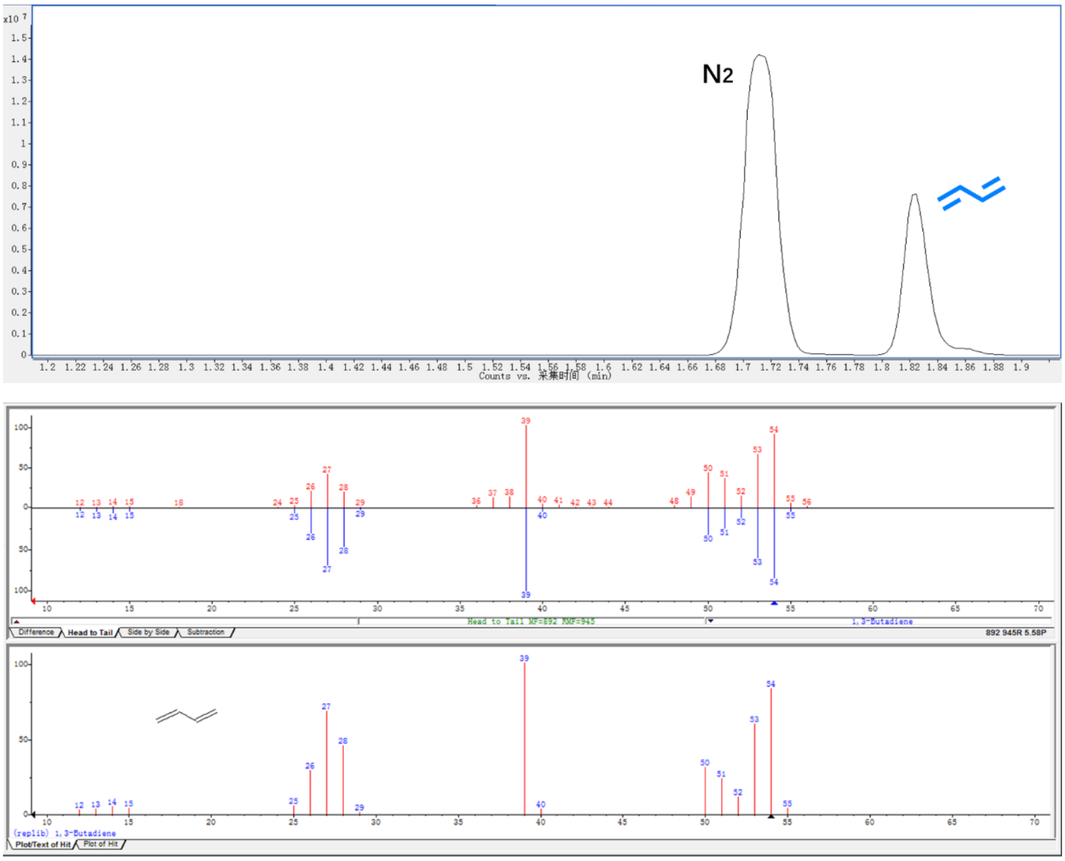


**Supplementary Figure 52. GC-MS spectrum of the gaseous products from PBT powder deconstruction over [$P_{4444}$]Br under the $N_2$ atmosphere.**

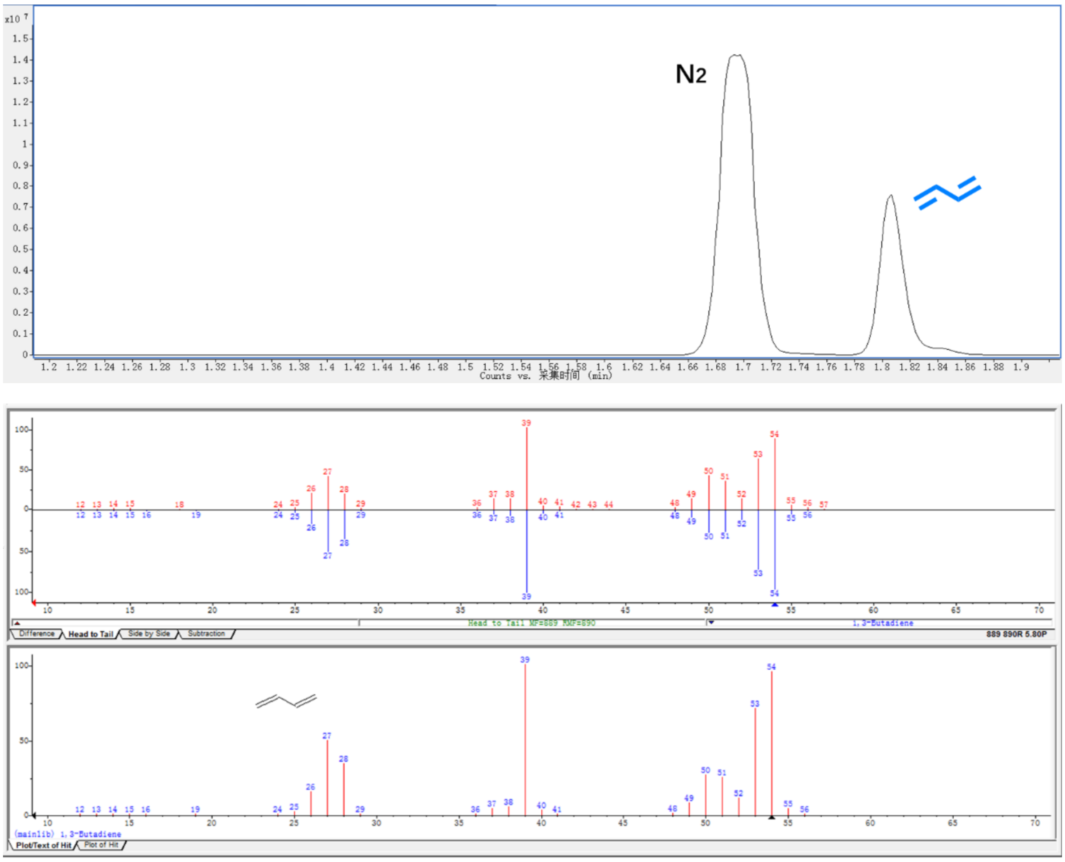


**Supplementary Figure 53. GC-MS spectrum of the gaseous products from PBS powder deconstruction over [$P_{4444}$]Br under the $N_2$ atmosphere.**

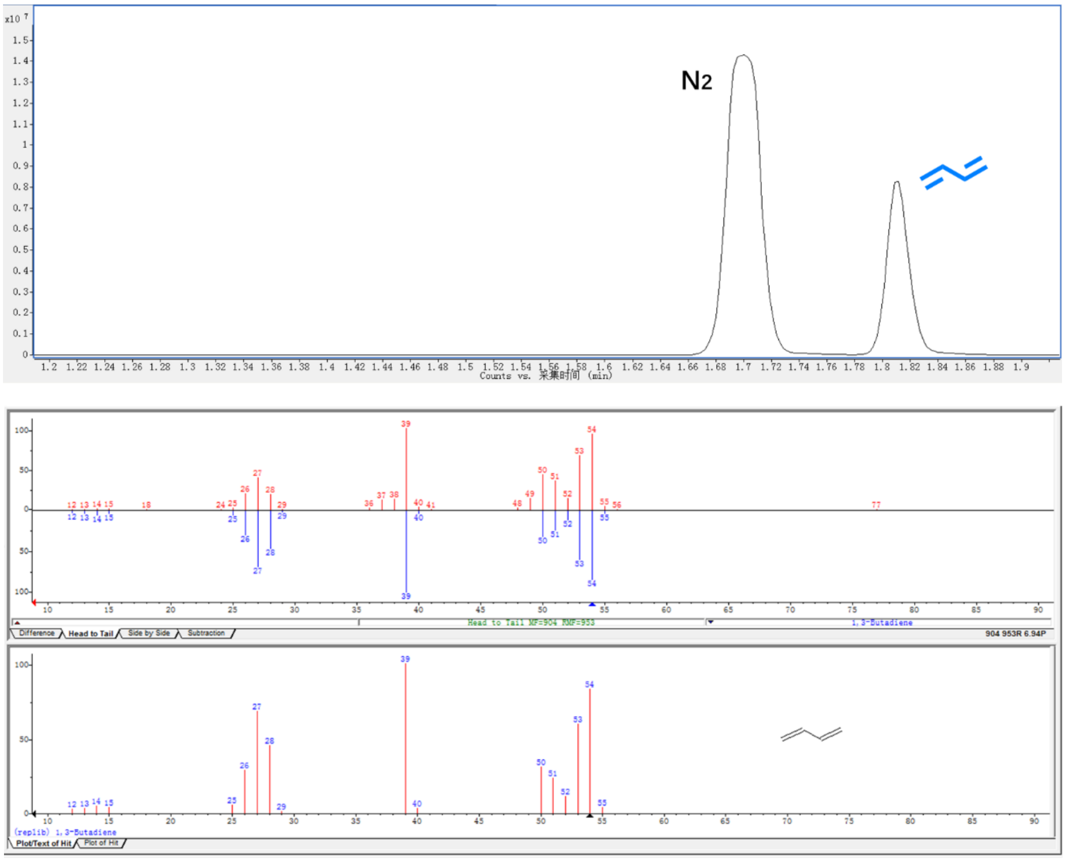


**Supplementary Figure 54. GC-MS spectrum of the gaseous products from PBA granule deconstruction over [P4444]Br under the $N_2$ atmosphere.**

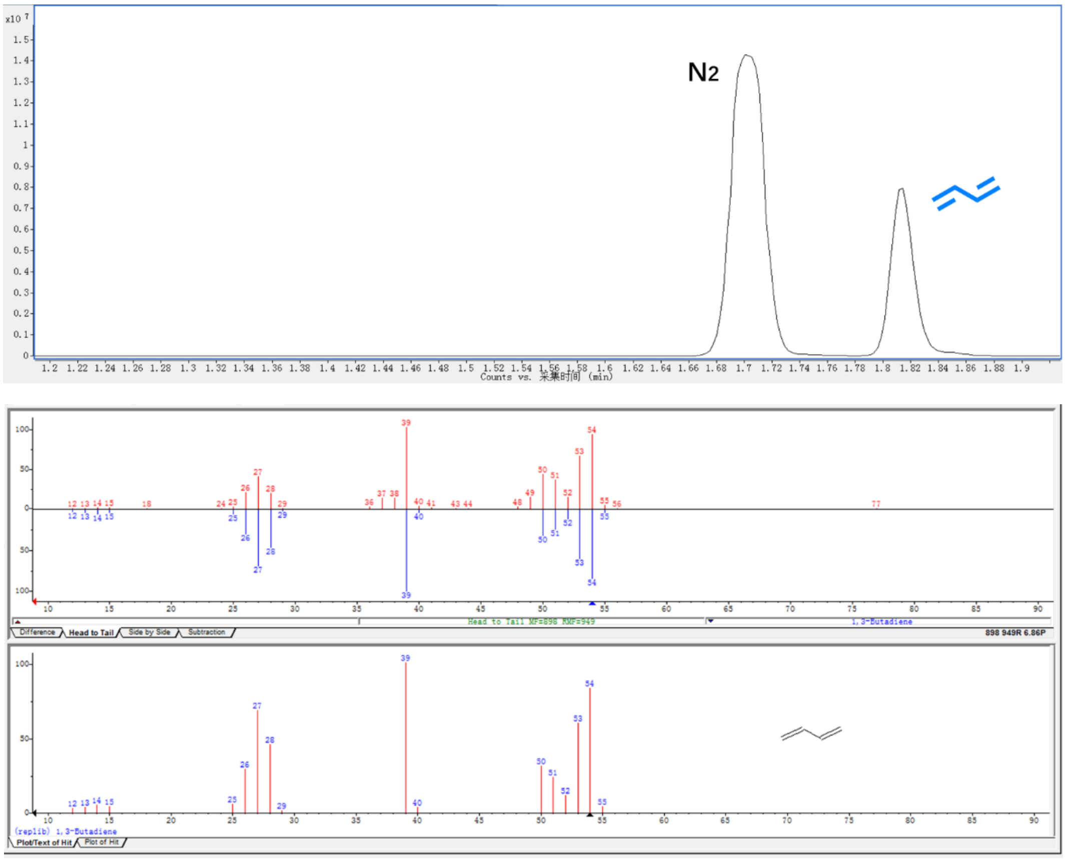


**Supplementary Figure 55. GC-MS spectrum of the gaseous products from PBAT granule deconstruction over [P$_{4444}$]Br under the N$_2$ atmosphere.**

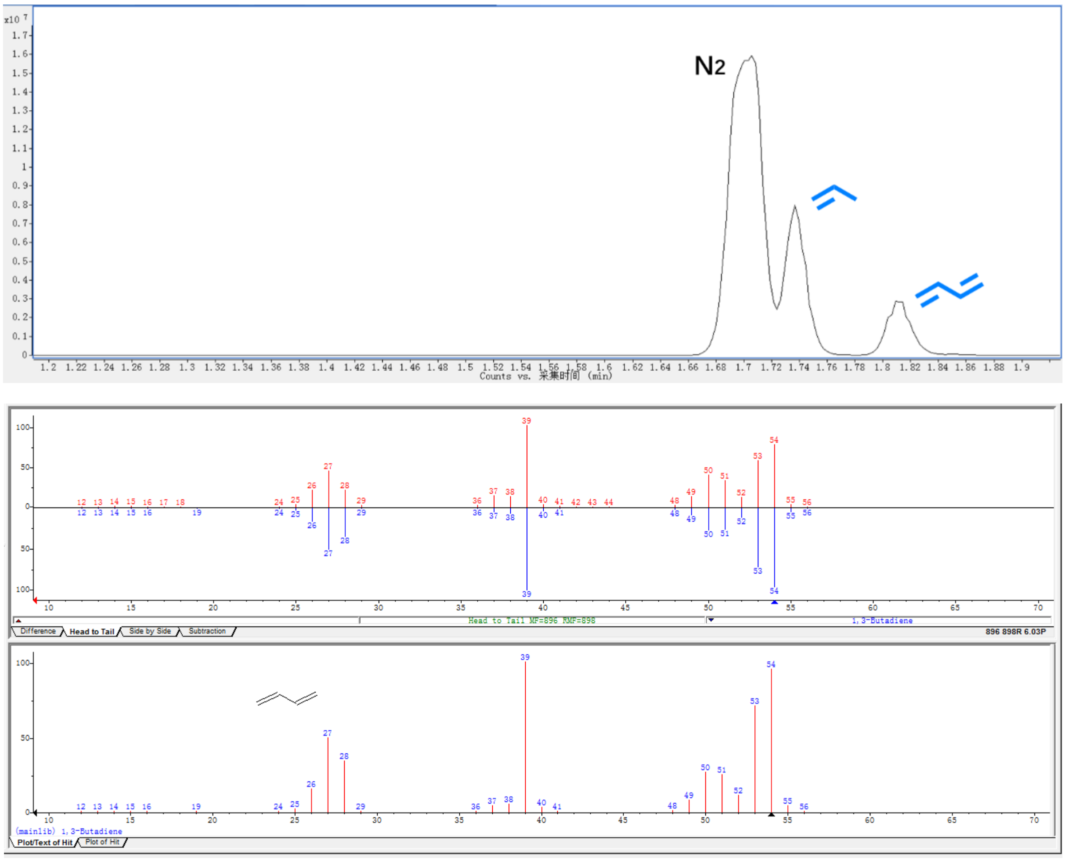


**Supplementary Figure 56. GC-MS spectrum of the gaseous products from the deconstruction for the mixture of PHB and PBT over [$P_{4444}$]Br under the $N_2$ atmosphere.**
